# Targeting DCLK1 overcomes 5‐fluorouracil resistance in colorectal cancer through inhibiting CCAR1/β‐catenin pathway‐mediated cancer stemness

**DOI:** 10.1002/ctm2.743

**Published:** 2022-05-06

**Authors:** Lanqing Wang, Lei Zhao, Zhenyu Lin, Dandan Yu, Min Jin, Pengfei Zhou, Jinghua Ren, Jing Cheng, Kunyu Yang, Gang Wu, Tao Zhang, Dejun Zhang

**Affiliations:** ^1^ Cancer Center Union Hospital Tongji Medical College Huazhong University of Science and Technology Wuhan 430022 China; ^2^ Wuhan YZY Medical Science & Technology Co., Ltd. Wuhan 430075 China

**Keywords:** cancer stemness, CCAR1, chemoresistence, colorectal cancer, DCLK1, β‐catenin

## Abstract

**Background:**

To date, 5‐fluorouracil‐based chemotherapy is very important for locally advanced or metastatic colorectal cancer (CRC). However, chemotherapy resistance results in tumor recurrence and metastasis, which is a major obstacle for treatment of CRC.

**Methods:**

In the current research, we establish 5‐fluorouracil resistant cell lines and explore the potential targets associated with 5‐fluorouracil resistance in CRC. Moreover, we perform clinical specimen research, in vitro and in vivo experiments and molecular mechanism research, to reveal the biological effects and the mechanism of DCLK1 promoting 5‐fluorouracil resistance, and to clarify the potential clinical value of DCLK1 as a target of 5‐fluorouracil resistance in CRC.

**Results:**

We discover that doublecortin‐like kinase 1 (DCLK1), a cancer stem cell maker, is correlated with 5‐fluorouracil resistance, and functionally promotes cancer stemness and 5‐fluorouracil resistance in CRC. Mechanistically, we elucidate that DCLK1 interacts with cell cycle and apoptosis regulator 1 (CCAR1) through the C‐terminal domain, and phosphorylates CCAR1 at the Ser343 site, which is essential for CCAR1 stabilisation. Moreover, we find that DCLK1 positively regulates β‐catenin signalling via CCAR1, which is responsible for maintaining cancer stemness. Subsequently, we prove that blocking β‐catenin inhibits DCLK1‐mediated 5‐fluorouracil resistance in CRC cells. Importantly, we demonstrate that DCLK1 inhibitor could block CCAR1/β‐catenin pathway‐mediated cancer stemness and consequently suppresses 5‐fluorouracil resistant CRC cells in vitro and in vivo.

**Conclusions:**

Collectively, our findings reveal that DCLK1 promotes 5‐fluorouracil resistance in CRC by CCAR1/β‐catenin pathway‐mediated cancer stemness, and suggest that targeting DCLK1 might be a promising method to eliminate cancer stem cells for overcoming 5‐fluorouracil resistance in CRC.

## Graphical abstract

1. DCLK1, a cancer stem cell maker, is correlated with 5‐fluorouracil resistance.
2. DCLK1 interacts with CCAR1 and phosphorylates CCAR1 at the Ser343 site, which is essential for CCAR1 stabilisation.
3. DCLK1 promotes 5‐fluorouracil resistance by CCAR1/β‐catenin pathway‐mediated cancer stemness.
4. Targeting DCLK1 might be a promising method to eliminate cancer stem cells for overcoming 5‐fluorouracil resistance in CRC.

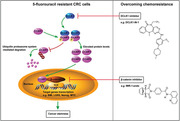

## 1 INTRODUCTION

Colorectal cancer (CRC) is the third most common cancer and second most common cause of cancer‐related deaths all over the world.^1^ In 2018, it has been estimated that over 1.8 million new CRC cases and 881 000 CRC‐related deaths occurred.^1^ In recent years, molecular targeted therapy (e.g. bevacizumab, cetuximab) and biological immunotherapy (e.g. immune checkpoint inhibitors) have made great progress. For instance, chemotherapy combing with bevacizumab^2^ or cetuximab^3^ improve the survival of metastatic CRC patients, and PD‐1 or CTLA4 inhibitors benefit metastatic CRC patients with d‐MMR/MSI‐H.^4^, ^5^ Nevertheless, 5‐fluorouracil‐based chemotherapy is still the cornerstone of treatment for locally advanced or metastatic CRC.^6^, ^7^ Tumour cells with intrinsical or acquired resistance to chemotherapy remains a challenge in clinical treatment, which consequently results in tumour recurrence and metastasis, and is the main cause of cancer‐related deaths. Hence, the mechanism of chemotherapy resistance desperately needs further researches to provide theoretical foundation for the clinical treatment.

Doublecortin‐like kinase 1 (DCLK1) was a microtubule‐associated protein and a member of the calmodulin‐dependent kinase (CaMK) family.^8^ Previous studies showed that DCLK1 was overexpressed in many tumours, including colorectal,^9^, ^10^, ^11^, ^12^, ^13^, ^14^, ^15^ pancreatic,^16^, ^17^ gastric,^18^ renal,^19^ hepatocellular carcinoma^20^ and other cancers.^21^, ^22^, ^23^, ^24^, ^25^, ^26^ The elevated levels of DCLK1 were closely correlated with poorer outcomes and tumour recurrence and metastasis.^27^ Moreover, growing evidences demonstrated critical functions of DCLK1 in tumour initiation, progression, epithelial‐mesenchymal transition (EMT) and cancer stemness.^28^, ^29^, ^30^ Most importantly, a series of studies have suggested the role of DCLK1 as a potential cancer stem cells (CSCs) marker in several tumours.^31^, ^32^, ^33^, ^34^ Westphalen et al.^32^ demonstrated DCLK1 marked quiescent progenitors that were candidates for pancreatic CSCs. Nakanishi et al.^33^ showed that DCLK1 marked CSCs of mouse intestinal adenomas by lineage‐tracing experiments. Nevi et al.^34^ identified DCLK1 as a putative novel stem cell marker, which characterised a specific CSCs subpopulation in human cholangiocarcinoma. Therefore, DCLK1 plays an essential role in maintaining cancer stemness.

It was well known that CSCs were resistant to chemoradiotherapy.^35^ Accumulating evidences had suggested that DCLK1, as a CSCs marker, was also a promising marker for chemoresistance.^36^, ^37^, ^38^ Such as, Panneerselvam et al.^37^ demonstrated that DCLK1 regulated the properties of CSC and cisplatin resistance in non‐small cell lung cancer via ABCD‐member‐4. Moreover, several studies also indicated that down‐regulating the expression or inhibiting the kinase activity of DCLK1 could improve the sensitivity to chemotherapy.^25^, ^39^, ^40^, ^41^ For instance, Zhang et al.^25^ demonstrated that knock‐down of DCLK1 attenuated tumourigenesis and improved the chemosensitivity of oesophageal squamous cell carcinoma by suppressing β‐catenin/c‐Myc signalling. Kawamura et al.^40^ demonstrated that LRRK2‐IN‐1 (a nonspecific selective inhibitor of DCLK1) enhanced the cytotoxic effects of gemcitabine via suppressing Chk1 phosphorylation in pancreatic cancer cells. Therefore, DCLK1 probably is a potential therapeutic target to overcome chemoresistance.^42^ However, the mechanisms of DCLK1‐promoting chemoresistance remains not fully understood. Especially, the relationship between DCLK1, chemoresistance and tumour stem cells needs to be further clarified.

In the current study, we discover that DCLK1 is correlated with 5‐fluorouracil resistance, and functionally promoted cancer stemness and 5‐fluorouracil resistance in CRC. Mechanistically, we elucidate that DCLK1 interacts with cell cycle and apoptosis regulator 1 (CCAR1) through the C‐terminal domain and phosphorylates CCAR1 at the Ser343 site, which is essential for CCAR1 stabilisation. Moreover, we find that DCLK1 positively regulates β‐catenin signalling via CCAR1, which is responsible for maintaining cancer stemness. Subsequently, we prove that blocking β‐catenin inhibits DCLK1‐mediated 5‐fluorouracil resistance in CRC cells. Therefore, our findings reveal that DCLK1 promotes 5‐fluorouracil resistance in CRC by CCAR1/β‐catenin pathway‐mediated cancer stemness. Importantly, we demonstrate that DCLK1 inhibitor could block CCAR1/β‐catenin pathway‐mediated cancer stemness and consequently suppresses 5‐fluorouracil resistant CRC cells in vitro and in vivo.

## 2 RESULTS

### 2.1 DCLK1 is correlated with 5‐fluorouracil resistance in CRC

**FIGURE 1:**
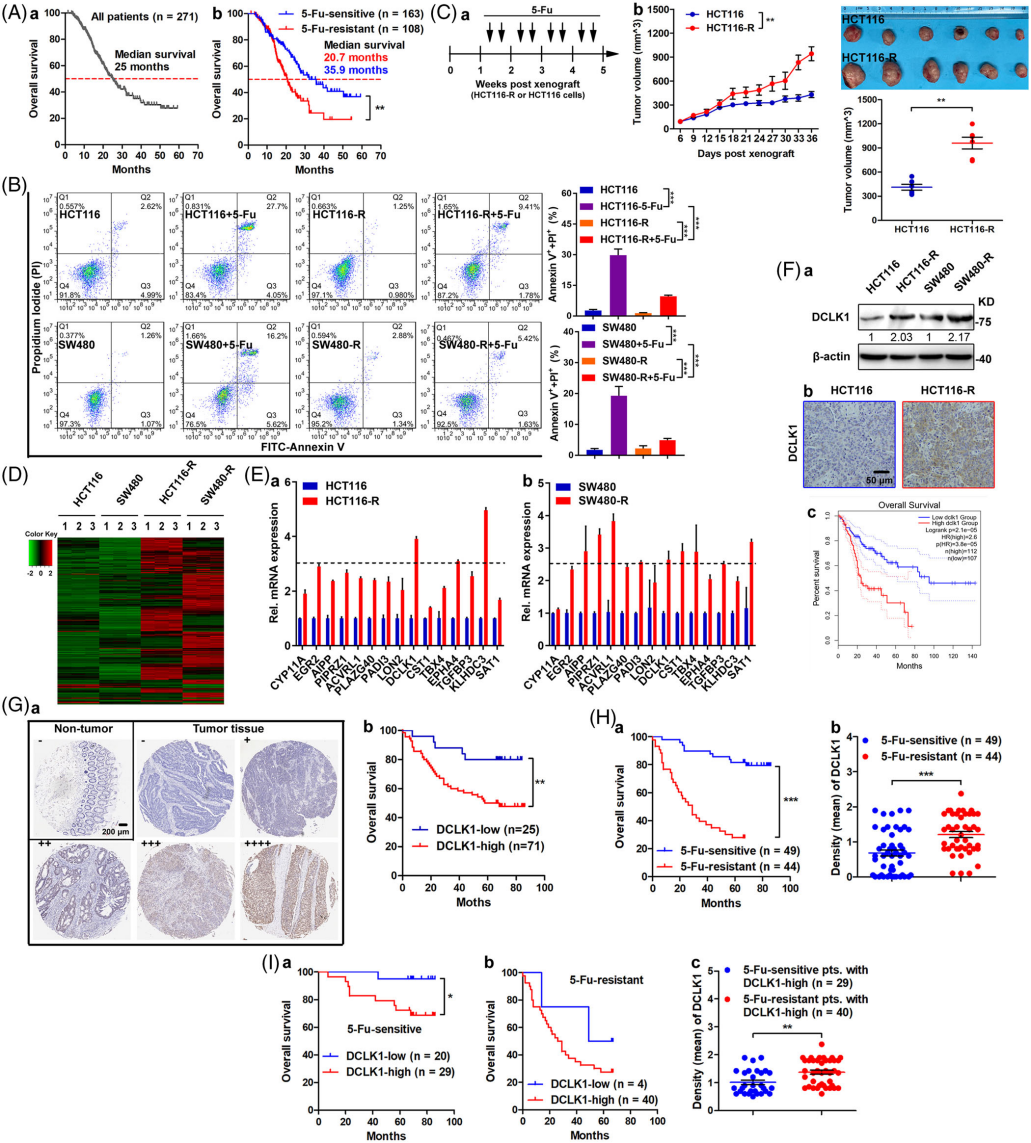
Doublecortin‐like kinase 1 (DCLK1) is correlated with 5‐fluorouracil resistance in colorectal cancer (CRC). (A) Kaplan–Meier plot of overall survival of CRC patients (*n* = 271) (a), and the 5‐fluorouracil sensitive patients (*n* = 163) and the 5‐fluorouracil resistant patients (*n* = 108) (b). (B) Apoptosis of 5‐fluorouracil resistant cells or parental cells. Cells were treated with 5‐fluorouracil for 48 h, then subjected to FITC‐annexin V/propidium iodide staining and analysed by FACS (*n* = 3). (C) The indicated cells were subcutaneously injected to nude mice, respectively (*n* = 6), then treated with 5‐fluorouracil twice a week for 3 weeks (a). The volumes of subcutaneous xenograft tumour were observed (b and c). (D) The genes differentially expressed in 5‐fluorouracil resistant cells or parental cells were detected by mRNA sequencing. (E) The interesting genes were verified by real‐time quantitative PCR (RT‐qPCR) (*n* = 3). (F) DCLK1 expression was analysed by Western blotting in the indicated cells and by immunohistochemistry in subcutaneous xenograft tumours. Survival analysis data were gained from the GEPIA2 database (http://gepia2.cancer‐pku.cn/#survival). (G) DCLK1 expression was analysed by immunohistochemistry in CRC tissues (*n* = 96). Kaplan–Meier plot of overall survival of CRC patients with high DCLK1 (*n* = 71) and low DCLK1 (*n* = 25). (H) Kaplan–Meier plot of overall survival and DCLK1 expression (immunohistochemical staining intensity) of 5‐fluorouracil sensitive (*n* = 49) and resistance (*n* = 44) patients. (I) Kaplan–Meier plot of overall survival of patients with DCLK1‐high or ‐low in 5‐fluorouracil sensitive and resistance patients, respectively. And DCLK1 expression (immunohistochemical staining intensity) of 5‐fluorouracil sensitive patients with DCLK1‐high (*n* = 29) and 5‐fluorouracil resistant patients with DCLK1‐high (*n* = 40) patients. Data are expressed as mean ± SD. *p < .05, **p < .01 and ***p < .001

It was well known that 5‐fluorouracil‐based chemotherapy was the cornerstone of treatment for locally advanced or metastatic CRC. In our hospital, 271 metastatic CRC patients who accepted 5‐fluorouracil‐based treatment were investigated, and the median overall survival (mOS) was 25 months (Figure 1A‐a). In subgroup analysis, 108 patients who developed progress disease during treatment or within 6 months after treatment were classified as resistant to treatment, while 163 patients were classified as sensitive to treatment. It was found that the mOS of 5‐fluorouracil resistant patients was shorter (20.7 months) than that of 5‐fluorouracil sensitive patients (35.9 months) (Figure 1A‐b). These data indicated that resistance to 5‐fluorouracil‐based chemotherapy result in poor outcomes of CRC patients.

To elucidate the mechanism of 5‐fluorouracil resistance and provide theoretical foundation for the clinical treatment, we established 5‐fluorouracil resistant cell lines (HCT116‐R and SW480‐R) as shown in Figure S1A, primary CRC cell lines (HCT116 and SW480) were treated with 5‐fluorouracil for over 8 months. Then the resistance of 5‐fluorouracil for these cell lines was confirmed. When treated with 5‐fluorouracil, the IC50 values of 5‐fluorouracil resistant cells were significantly higher than the respective parental cells (Figure S1B), and the apoptosis of 5‐fluorouracil resistant cells was notably decreased compared to the respective parental cells (Figure 1B). Moreover, in subcutaneous xenograft tumour models treated with 5‐fluorouracil, data showed that the volumes of subcutaneous tumours in 5‐fluorouracil resistant group were significantly greater compared to the parental group (Figure 1C). Collectively, these established 5‐fluorouracil resistant cell lines were proved to be resistant to 5‐fluorouracil in vitro and in vivo.

To explore the potential targets associated with 5‐fluorouracil resistance in CRC, mRNA sequencing was applied to detect differentially expressed genes (Figure 1D). There were 594 up‐regulated genes in both of HCT116‐R and SW480‐R cells compared to the parental cells (Figure S1C), and the top 15 genes were screened for verification (Figure 1E). In contrast with the parental cells, genes encoding protein kinases and up‐regulated over twofold were our interesting targets. Then *DCLK1, PADIA3* and *EPHA4* were chosen to be investigated. Further verification and bio‐informatics analysis were conducted, data showed that DCLK1 was significantly up‐regulated in 5‐fluorouracil resistant cells and in subcutaneous tumour with 5‐fluorouracil resistance (Figure 1F‐a,b), and survival analysis from the GEPIA2 database showed that patients with high DCLK1 had poorer overall survival than patients with low DCLK1 (Figure 1F‐c). Importantly, knock‐down of DCLK1 sensitised CRC cells to 5‐fluorouracil. However, PADIA3 or EPHA4 performed poorly (Figure S1D–F). Therefore, DCLK1 was identified as a candidate target gene for further analysis.

Accumulating evidences showed that DCLK1 was overexpressed in several types of tumours, especially in CRC^9^, ^10^, ^11^, ^12^, ^13^, ^14^, ^15^ and pancreatic cancer.^16^, ^17^ And elevated levels of DCLK1 were closely correlated with poorer outcomes and higher rates of tumour recurrence and metastasis.^27^ In consistent with these studies, we also found that DCLK1 was highly expressed in CRC tissues, and patients with high DCLK1 had poorer overall survival compared with low DCLK1 (Figure 1G). Moreover, in subgroup analysis, patients who were resistant to 5‐fluorouracil‐based treatment had shorter overall survival compared with patients who were sensitive to treatment (Figure 1H‐a). Importantly, DCLK1 expression in patients who were resistant to treatment was higher than patients who were sensitive to treatment (Figure 1H‐b). Interestingly, regardless of patients who were treatment sensitive or not, patients with high DCLK1 expression had shorter overall survival compared with low DCLK1 (Figure 1I‐a,b). In addition, DCLK1 expression in 5‐fluorouracil resistant patients with high DCLK1 was greater than in 5‐fluorouracil sensitive patients with high DCLK1 (Figure 1I‐c). As a whole, these results highlighted a correlation between DCLK1 and 5‐fluorouracil resistance.

### 2.2 DCLK1 promotes cancer stemness and 5‐fluorouracil resistance in CRC

**FIGURE 2:**
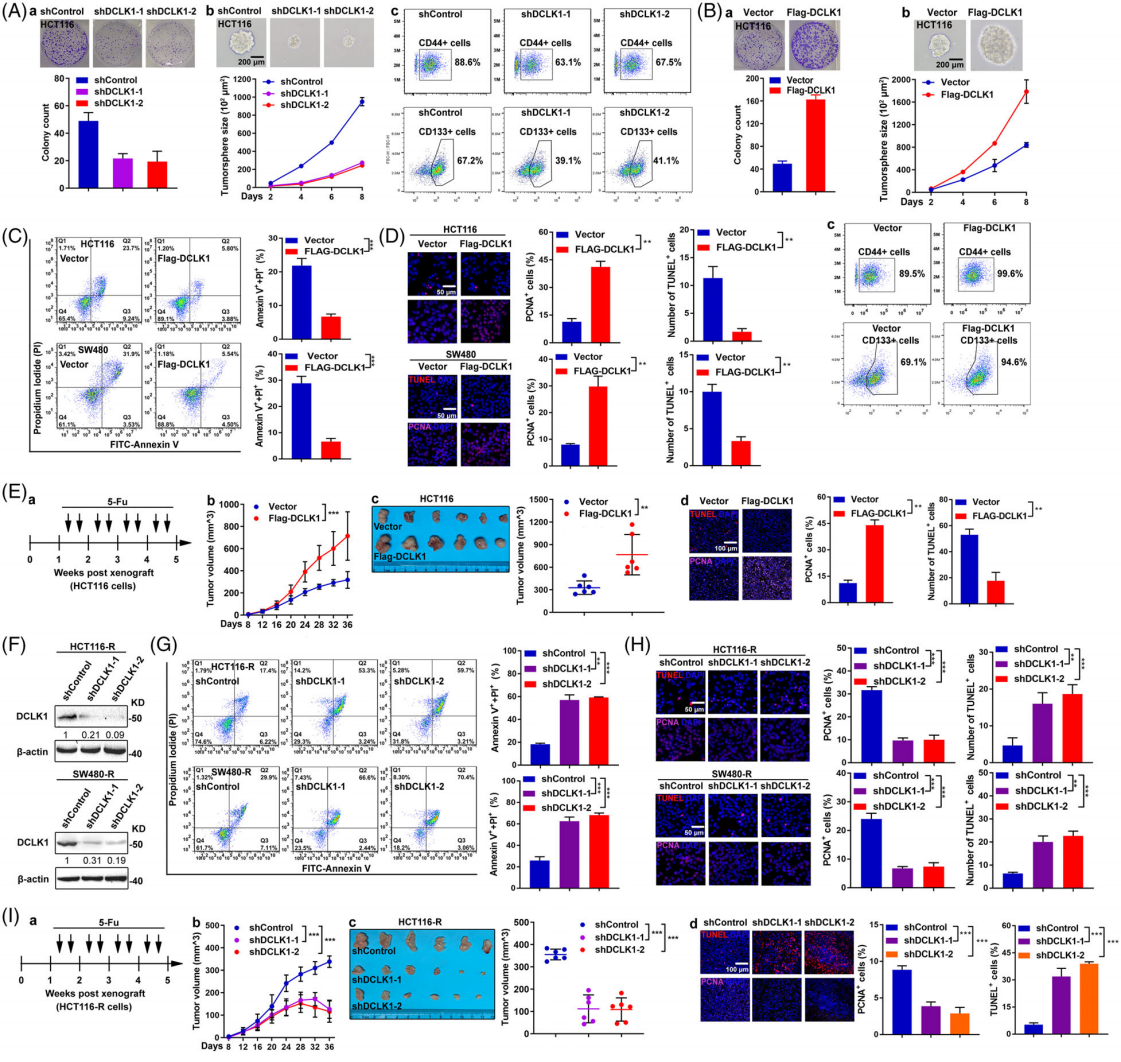
Doublecortin‐like kinase 1 (DCLK1) promotes cancer stemness and 5‐fluorouracil resistance in colorectal cancer (CRC). (A) Colony formation ability and self‐renewal activity of HCT116 cells transfected with the indicated shRNAs was determined by clonogenic assay (a) and tumoursphere‐forming assay respectively (b) (*n* = 3). Scale bar represents 200 μm. CD44+ and CD133+ cells were detected by FACS assay (c). (B) Colony formation ability and self‐renewal activity of HCT116 cells transfected with the indicated constructs was determined by clonogenic assay (a) and tumoursphere‐forming assay, respectively (b) (*n* = 3). Scale bar represents 200 μm. CD44+ and CD133+ cells were detected by FACS assay (c). (C) Apoptosis of HCT116 and SW480 cells transfected with the indicated constructs and treated with 5‐fluorouracil for 48 h, were subjected to FITC‐annexin V/propidium iodide staining and analysed by FACS (*n* = 3). (D) TUNEL staining (red) PCNA staining (purple) in HCT116 and SW480 cells. Cells were transfected with the indicated constructs and treated with 5‐fluorouracil for 48 h, then subjected to immunofluorescence staining. (E) HCT116 stable cell lines transfected with the indicated constructs were subcutaneously injected to nude mice, respectively (*n* = 6), then treated with 5‐fluorouracil twice a week for 3 weeks (a). The volumes of subcutaneous xenograft tumour were observed (b and c), and tumours were analysed by TUNEL staining (red) and PCNA staining (purple) (d). (F) Western blotting showed the knock‐down efficiency of DCLK1 by two different shRNAs in 5‐fluorouracil resistant cells (HCT116‐R and SW480‐R). (G) Apoptosis of HCT116‐R and SW480‐R cells transfected with the indicated shRNAs for 48 h and treated with 5‐fluorouracil for 24 h, then were subjected to FITC‐annexin V/propidium iodide staining and analysed by FACS (*n* = 3). (H) TUNEL staining (a maker of apoptosis, red) and PCNA staining (a maker of proliferation, purple) of HCT116‐R and SW480‐R cells. Cells were transfected with the indicated shRNAs and treated with 5‐fluorouracil for 48 h, then subjected to immunofluorescence staining. (I) HCT116 stable cell lines transfected with the indicated shRNAs were subcutaneously injected to nude mice, respectively (*n* = 6), then treated with 5‐fluorouracil twice a week for 3 weeks (a). The volumes of subcutaneous xenograft tumour were observed (b and c), and tumours were analysed by TUNEL staining (red) and PCNA staining (purple) (d). Data are expressed as mean ± SD. *p < .05, **p < .01 and ***p < .001. PCNA, proliferating cell nuclear antigen; TUNEL, TdT‐mediated dUTP Nick‐End Labeling

Mounting evidences had disclosed that DCLK1 was a CSC maker in many tumours, including pancreatic cancer^32^ and CRC,^31^, ^33^ and DCLK1‐induced cancer stemness was essential to tumour initiation and progression.^28^, ^29^, ^30^ In consistent with these studies, we found that knock‐down of DCLK1 resulted in the weakened proliferative potential and self‐renewal ability of CRC cells, and the decreased proportion of CD44+ and CD133+ CRC cells (Figures 2A and S2A,B). In contrast, exogenously expressed DCLK1 led the proliferative potential and the self‐renewal ability of CRC cells to be enhanced, and the proportion of CD44+ and CD133+ CRC cells to be increased (Figures 2B and S2C,D). Moreover, data showed that, the weakened proliferative potential and self‐renewal ability, and the decreased proportion of CD44+ and CD133+ CRC cells mediated by down‐regulation of DCLK1, could be rescued by DCLK1 exogenous expression (Figure S3). These results indicated a critical role of DCLK1 in maintaining cancer stemness of CRC cells.

CSCs were mainly responsible for chemoradiotherapy resistance, relapse of tumour and metastatic behaviours.^35^ We hypothesised that DCLK1, a CSCs marker, played an essential role in 5‐fluorouracil resistance. Then we assessed the influence of DCLK1‐overexpression on the sensitivity of primary CRC cells to 5‐fluorouracil. Data showed that, when treated with 5‐fluorouracil, the apoptosis (Figure 2C) and the TdT‐mediated dUTP Nick‐End Labeling (TUNEL) staining (a maker of apoptosis) of DCLK1‐overexpressing CRC cells were significantly decreased, while the proliferating cell nuclear antigen (PCNA) staining (a maker of proliferation) was notably increased (Figure 2D). Moreover, in subcutaneous xenograft tumour models treated with 5‐fluorouracil, the volumes of subcutaneous xenograft tumour in DCLK1‐overexpressing group were significantly greater compared to the control (Figure 2E‐a–c), and the TUNEL staining of subcutaneous xenograft tumour was decreased, while the PCNA staining was increased (Figure 2E‐d). Meanwhile, we evaluated the influence of DCLK1 knock‐down on the sensitivity of 5‐fluorouracil resistant CRC cells to 5‐fluorouracil. Data showed that, when treated with 5‐fluorouracil, the apoptosis (Figure 2G) and the TUNEL staining of 5‐fluorouracil resistant cells with DCLK1 knock‐down were significantly increased, while the PCNA staining was markedly decreased (Figure 2H). Moreover, in subcutaneous xenograft tumour models treated with 5‐fluorouracil, the volumes of subcutaneous xenograft tumour in DCLK1 knock‐down group were significantly smaller compared to the control (Figure 2I‐a–c), and the TUNEL staining of subcutaneous xenograft tumour was increased, while the PCNA staining was decreased (Figure 2I‐d). Collectively, these findings underlined that DCLK1 promoted cancer stemness and 5‐fluorouracil resistance in CRC.

### 2.3 Identifying the C‐terminal domain of DCLK1 to interact with CCAR1

**FIGURE 3:**
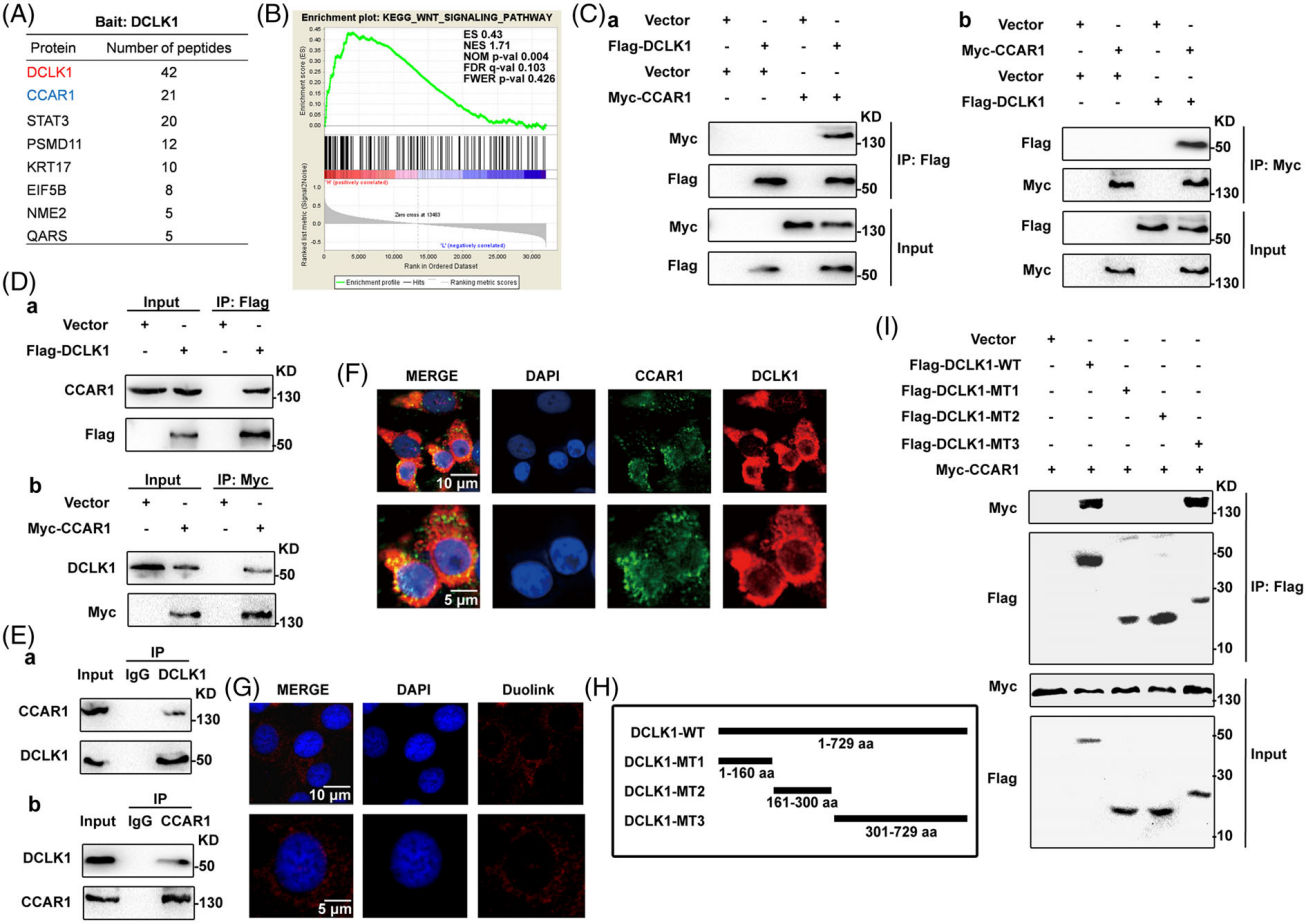
Identifying the C‐terminal domain of doublecortin‐like kinase 1 (DCLK1) to interact with cell cycle and apoptosis regulator 1 (CCAR1). (A) Tandem Affinity Purification‐Mass Spectrometry analysis showed DCLK1‐binding proteins in HEK293T cells. The number of peptides for each protein identified was listed. (B) Gene Set Enrichment Analysis (GSEA) of single gene showed that DCLK1 was positively correlated to Wnt/β‐catenin signal pathway. The original mRNA expression data were gained from the TCGA database (https://portal.gdc.cancer.gov/projects/TCGA‐COAD), and the gene set pathway data were gained from the enrichment pathway collection of the KEGG database on the GSEA data analysis website (http://www.gsea‐msigdb.org/gsea/login.jsp). (C and D) HEK293T cells co‐transfected with Flag‐DCLK1 and Myc‐CCAR1 constructs for 24 h were lysed with NP40 buffer. Then cell lysates were collected and analysed by immunoprecipitation using S‐protein agarose beads or Myc agarose and Western blotting with the indicated antibodies (*n* = 3). (E) HCT116 cells were collected and conducted to immunoprecipitation using anti‐DCLK1 or anti‐CCAR1 antibodies, and then analysed by Western blotting (*n* = 3). (F) Immunofluorescence staining determined the location of DCLK1 (red) and CCAR1 (green) proteins. (G) The Duolink PLA probe experiment detected the interaction between DCLK1 and CCAR. (H) Schematic description of wild‐type DCLK1 (DCLK1‐WT) and DCLK1 deletion mutants (DCLK1‐MT) used in this study. (I) HEK293T cells co‐transfected with the indicated constructs encoding Flag‐DCLK1 and indicated CCAR1 mutants for 24 h were collected and lysed. Then the supernatants were conducted to immunoprecipitation using S‐protein agarose beads, and analysed by Western blotting with the indicated antibodies (*n* = 3). KEGG, Kyoto Encyclopedia of Genes and Genomes; TCGA, The Cancer Genome Atlas

To elucidate the involved mechanism of DCLK1‐mediated cancer stemness and 5‐fluorouracil resistance in CRC, we performed mass spectrometry (MS) to identify DCLK1‐binding proteins in cells (Figure 3A). The Gene Set Enrichment Analysis (GSEA) showed that, there was a positive correlation between DCLK1 expression and Wnt/β‐catenin signal pathway (Figure 3B). It was reported that CCAR1 was a regulator of cell proliferation as well as apoptosis signalling, which interacted with β‐catenin and enhanced the ability of β‐catenin to activate expression of Wnt/β‐catenin target genes.^43^ Hence, these data uncovered CCAR1 as the promising candidate of DCLK1‐interacting protein. To validate the above theory, we conducted the following co‐immunoprecipitation experiments. As shown in Figure 3C, exogenous DCLK1 was able to bind to exogenous CCAR1, and vice versa. Moreover, we also showed that exogenous DCLK1 interacted with endogenous CCAR1, and vice versa (Figure 3D). Furthermore, the endogenous interaction between DCLK1 and CCAR1 was observed in CRC cells (Figure 3E). Mostly, we found that DCLK1 and CCAR1 were mainly co‐located in the cytoplasm (Figure 3F), and the Duolink PLA Probe experiment further confirmed an interaction between DCLK1 and CCAR1 in the cytoplasm (Figure 3G). Therefore, these results strongly suggested that DCLK1 physically interacted with CCAR1 directly.

DCLK1 harbours two double cortin (DCX) domains in the N terminus, a serine/threonine protein kinase domain in the C terminus and a serine/proline rich domain between the N and C terminus.^8^ To further investigate which domain of DCLK1 was required for binding to CCAR1, we generated three DCLK1 deletion mutants with a C‐terminal Flag tag, as indicated in Figure 3H. Each of DCLK1 mutants and CCAR1 transcript were co‐expressed in HEK293T cells, respectively. Then the co‐immunoprecipitation assay was performed using the whole cell lysates. As illustrated in Figure 3I, DCLK1‐MT3 (amino acid 301–729) was observed to bind to CCAR1, rather than DCLK1‐MT1 (amino acid 1–160) and DCLK1‐MT2 (amino acid 161–300). Collectively, these results demonstrated that the amino acid sequence spanning Pro301 to Met729 in the C‐terminal domain of DCLK1 was indispensable for binding to CCAR1.

### 2.4 DCLK1 positively regulates the stability of CCAR1

**FIGURE 4:**
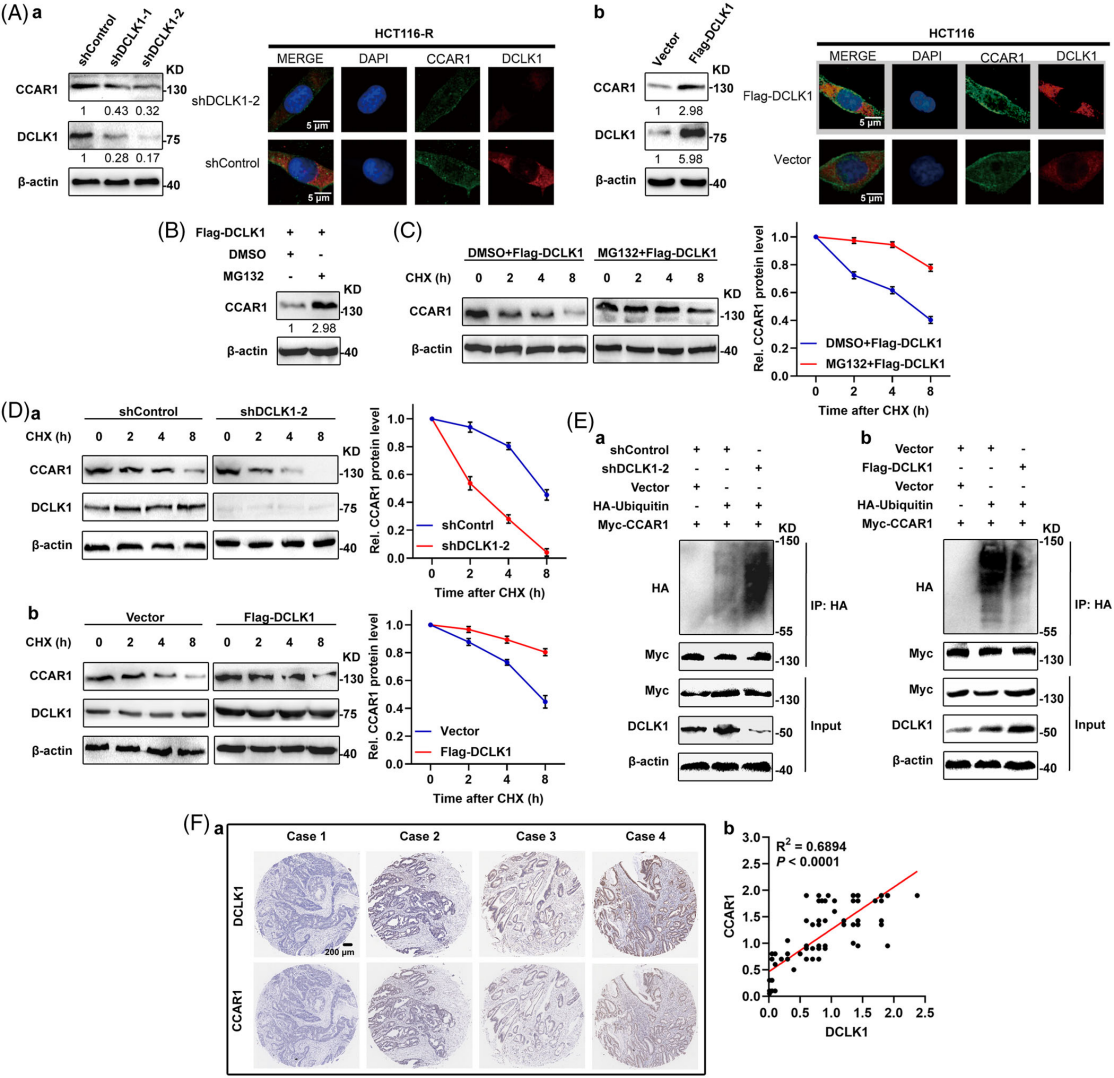
Doublecortin‐like kinase 1 (DCLK1) positively regulates the stability of cell cycle and apoptosis regulator 1 (CCAR1). (A) The protein levels of DCLK1 and CCAR1. HCT116 cells were transfected with the indicated shRNAs or constructs for 48 h, and cell lysates were collected and analysed by Western blotting with the indicated antibodies (*n* = 3). Immunofluorescence staining was used to detect the expression of protein of DCLK (red) and CCAR1 (green). (B) HCT116 cells transfected with the indicated constructs were treated with or without MG132. Then whole cell lysates were collected and analysed by Western blotting (*n* = 3). (C) The half‐life of CCAR1. Left panel: HCT116 cells were transfected with the indicated constructs and harvested at the indicated points after cycloheximide (CHX) with or without MG132 treatment for Western blotting analysis. Right panel: quantification of the CCAR1 band intensity was shown (*n* = 3). (D) The half‐life of CCAR1. Left panel: HCT116 cells were transfected with the indicated shRNAs or constructs for 48 h. Cells were treated with 20 μg/ml of CHX and harvested at the indicated time points. The protein level of CCAR1 was detected by Western blotting. Right panel: quantification of the CCAR1 band intensity was shown (*n* = 3). (E) The ubiquitination of CCAR1. HEK293T cells transfected with the indicated shRNAs or constructs for 24 h. Cells were treated with MG132 (10 μM) for 4 h before harvesting. The lysates were incubated with Myc beads and then subjected to Western blotting (*n* = 3). (F) Representative immunohistochemical staining of DCLK1 and CCAR1 in the paired colorectal cancer (CRC) tissues (a). Linear regression analysis of the expression of DCLK1 and CCAR1 (b). Data are expressed as mean ± SD

Having proved a physical interaction between these two proteins, we next evaluated the effect of DCLK1 on CCAR1. As shown in Figures 4A and S4A, knock‐down of DCLK1 dramatically resulted in the decrease of endogenous CCAR1, while exogenously expressed DCLK1 resulted in the accumulation of endogenous CCAR1. Interestingly, no obvious variations were observed between the mRNA levels of CCAR1 when DCLK1 was up or down‐regulated (Figure S4B). Moreover, treatment with MG132 (proteasome inhibitor) led to increased CCAR1 protein levels (Figure 4B), which indicated the involvement of the ubiquitin/proteasome system in controlling the stability of CCAR1. In line with the above observation, the half‐life of CCAR1 was markedly prolonged after MG132 treatment (Figure 4C). Furthermore, we found that the half‐life of endogenous CCAR1 protein was notably shortened in DCLK1 knock‐down cells and was extended in DCLK1‐overexpression cells when compared to that of control cells (Figure 4E). Ubiquitination assays showed that DCLK1 knock‐down led to increased CCAR1 poly‐ubiquitination, while exogenously expressed DCLK1 led to reduced CCAR1 poly‐ubiquitination (Figure 4D). In addition, to further validate the correlation between DCLK1 and CCAR1, we detected the protein levels of DCLK1 and CCAR1 in CRC tissues by immunohistochemistry (IHC). It showed that the expression of DCLK1 was positively correlated with that of CCAR1 (Figure 4F). Collectively, these results suggested that DCLK1 positively regulated the stability of CCAR1 via the ubiquitin–proteasome pathway.

### 2.5 DCLK1 phosphorylates CCAR1 at Ser343 site which is essential for CCAR1 stabilisation

**FIGURE 5:**
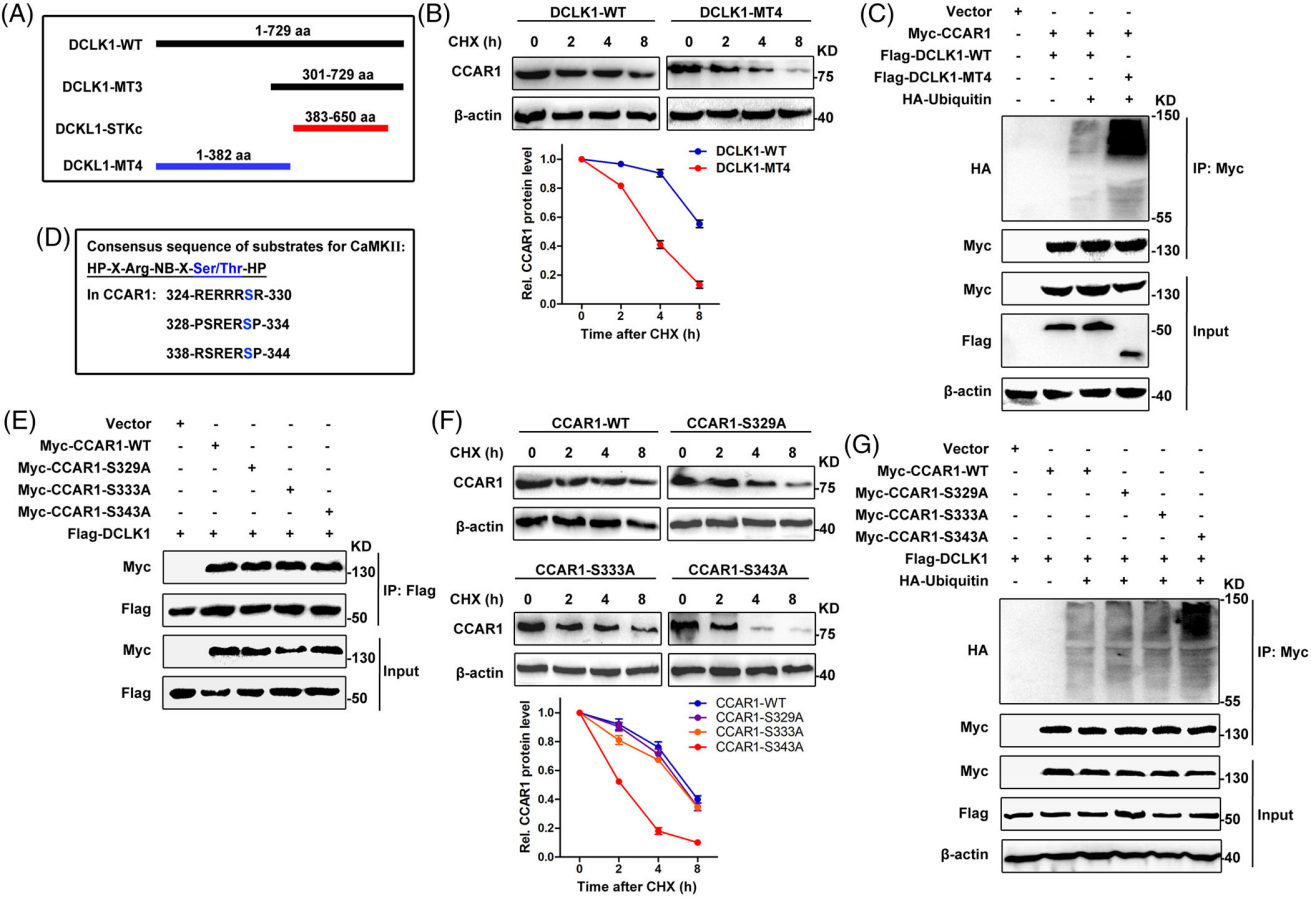
Doublecortin‐like kinase 1 (DCLK1) phosphorylates cell cycle and apoptosis regulator 1 (CCAR1) at Ser343 site which is essential for CCAR1 stabilisation. (A) Schematic description of a catalytic domain of the serine/threonine kinase in the C‐terminal of DCLK1. (B) The half‐life of CCAR1. Up panel: HCT116 cells were transfected with the indicated constructs for 48 h. Cells were treated with 20 μg/ml of cycloheximide (CHX) and harvested at the indicated time points. The protein level of CCAR1 was detected by Western blotting. Down panel: quantification of the CCAR1 band intensity was shown. (C) The ubiquitination of CCAR1. HEK293T cells were transfected with the indicated constructs for 24 h, then treated with MG132 (10 μM) for 4 h before harvesting. The lysates were incubated with Myc beads and then subjected to Western blotting. (D) The conserved HP‐X‐Arg‐NB‐X‐Ser/Thr‐HP (R‐NB‐X‐S/T‐HP) motif in substrates for CaMKII (X, NB and HP represent any amino acid, non‐basic and hydrophobic amino acids, respectively, and Ser/Thr denote sites of phosphorylation). And the candidate serine sites in CCAR1. (E) HEK293T cells were transfected with the indicated constructs for 24 h were collected and then conducted to immunoprecipitation. These samples were analysed by Western blotting with the indicated antibodies. (F) The half‐life of wild‐type CCAR1 and CCAR1 mutants. Up panel: HCT116 cells were transfected with the indicated constructs for 48 h. Cells were treated with 20 μg/ml of CHX and harvested at the indicated time points. The protein level of CCAR1 was detected by Western blotting. Down panel: quantification of the CCAR1 band intensity was shown. (G) The ubiquitination of wild‐type CCAR1 and CCAR1 mutants. HEK293T cells were transfected with the indicated constructs for 24 h, then treated with MG132 (10 μM) for 4 h before harvesting. The lysates were incubated with Myc beads and then subjected to Western blotting. Data are expressed as mean ± SD

DCKL1 contained a catalytic domain of the serine/threonine kinase with similarity to CaMKs in the C‐terminal amino acid 383–650^44^ (Figure 5A). Considering that the C‐terminal domain (amino acid 301–729) of DCLK1 was indispensable for binding to CCAR1, and DCLK1 positively regulated the stability of CCAR1, we speculated that the DCLK1‐dependent CCAR1 stabilisation required its ability to phosphorylate CCAR1. To confirm that, we generated a DCLK1 deletion mutant (amino acid 1–382) without C‐terminal catalytic domain (DCLK1‐MT4), as indicated in Figure 5A. We found that DCLK1 mutant without C‐terminal catalytic domain led the half‐life of CCAR1 to be shorted (Figure 5B) and the ubiquitination of CCAR1 to be increased (Figure 5C) compared to wild‐type DCLK1. Therefore, these findings indicated that C‐terminal catalytic domain of DCLK1 was indispensable for CCAR1 stabilisation.

It was well known that most CaMKs substrates contained a conserved Arg‐NB‐X‐Ser/Thr‐HP (R‐NB‐X‐S/T‐HP) motif.^45^ Interestingly, we found that there were three serine sites in CCAR1 that contain R‐NB‐X‐S/T‐HP motif, as shown in Figure 5D. However, there was no specific antibody for detecting these phosphorylation sites in CCAR1, we could not directly examine the changes of phospho‐CCAR1‐S329, ‐S333 and ‐S343 levels upon DCLK1 expression. Then we assessed whether DCLK1 binding to CCAR1, or DCLK1‐mediated CCAR1 stability were affected by the above CCAR1 serine sites. As shown in Figure 5E, like wild‐type CCAR1 (CCAR1‐WT), all of the mutants CCAR1‐S329A, ‐S333A and ‐S343A were interacted with DCLK1, suggesting that these serine sites in CCAR1 were not responsible for DCLK1 binding to CCAR1. Notably, the protein degradation assay showed that, the half‐life of the mutant CCAR1‐S343A was remarkably shorter than that of the CCAR1‐WT, the mutants CCAR1‐S329A and ‐S333A upon DCLK1 expression (Figure 5F). Moreover, ubiquitination assay demonstrated that, the ubiquitination of the mutant CCAR1‐S343A was substantially increased compared to that of CCAR1‐WT and the mutants CCAR1‐S329A and ‐S333A upon DCLK1 expression (Figure 5G). Data supported that the Ser343 site in a conserved R‐NB‐X‐S/T‐HP motif was responsible for DCLK1‐mediated CCAR1 stability. Taken together, these results uncovered that DCLK1 can phosphorylate CCAR1 at Ser343 site, which was essential for CCAR1 stabilisation.

### 2.6 DCLK1 positively regulates β‐catenin signalling via CCAR1

It was reported that CCAR1 interacts with β‐catenin and enhanced the ability of β‐catenin to activate expression of Wnt/β‐catenin target genes.^43^ Our results had suggested that DCLK1 interacted with CCAR1 and positively regulated the stability of CCAR1, and GSEA data showed that DCLK1 was positively correlated to Wnt/β‐catenin pathway. Therefore, we hypothesised that DCLK1 promoted β‐catenin signalling via CCAR1.

**FIGURE 6:**
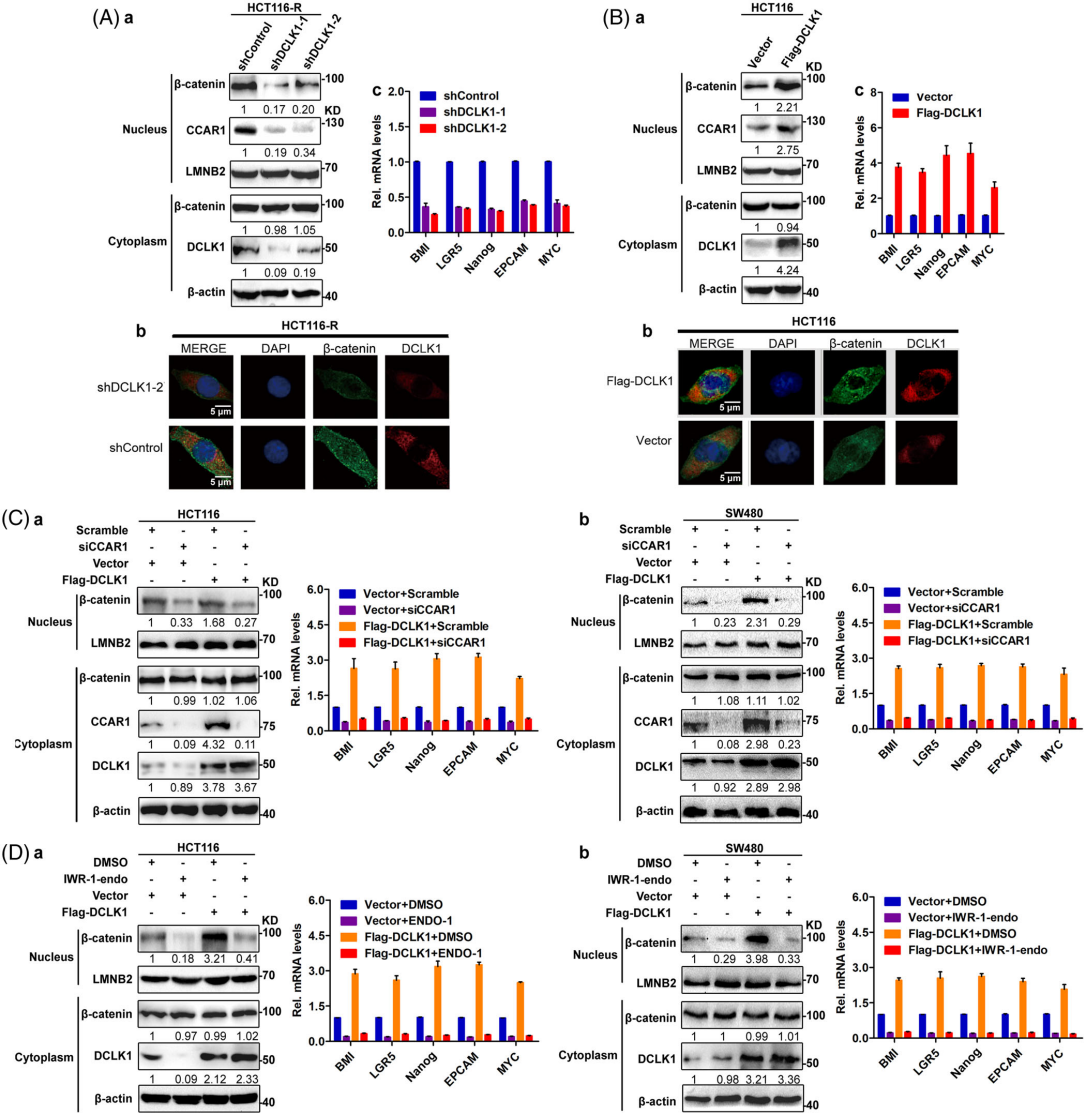
Doublecortin‐like kinase 1 (DCLK1) positively regulates β‐catenin signalling via cell cycle and apoptosis regulator 1 (CCAR1). (A) HCT116‐R cells were transfected with the indicated shRNAs for 48 h. Protein in nucleus or cytoplasm, and total RNA were collected. Western blotting was conducted to detect the indicated protein level, and real‐time quantitative PCR (RT‐qPCR) was performed to analyse the expression of β‐catenin target genes (*n* = 3). Immunofluorescence staining was used to detect the expression of DCLK (red) and β‐catenin (green). (B) HCT116 cells were transfected with the indicated constructs for 48 h. Protein in nucleus or cytoplasm, and total mRNA were collected. Western blotting was conducted to detect the indicated protein level, and RT‐qPCR was performed to analyse the expression of β‐catenin target genes (*n* = 3). Immunofluorescence staining was used to detect the expression of DCLK (red) and β‐catenin (green). (C) HCT116 and SW480 cells were co‐transfected with the indicated constructs and siRNAs for 48 h. Protein in nucleus or cytoplasm, and total RNA were collected. Western blotting was conducted to detect the indicated protein level, and RT‐qPCR was performed to analyse the expression of β‐catenin target genes (*n* = 3). (D) HCT116 and SW480 cells were transfected with the indicated constructs for 48 h, then treated with β‐catenin inhibitor (IWR‐1‐endo) for 24 h. Protein in nucleus or cytoplasm, and total RNA were collected. Western blotting was conducted to detect the indicated protein level, and RT‐qPCR was performed to analyse the expression of β‐catenin target genes (*n* = 3). Data are expressed as mean ± SD

We found that knock‐down of DCLK1 dramatically decreased the protein level of β‐catenin and CCAR1 in nucleus (but not in cytoplasm), and the expression of β‐catenin target genes (Figures 6A and S5A), which were implicated in regulation of CSC (e.g. proto‐oncogene BMI1, LGR5, Nanog), EMT (e.g. Epithelial Cell Adhesion Molecule) and cell proliferation (e.g. proto‐oncogene MYC). While exogenously expressed DCLK1 increased the protein level of β‐catenin and CCAR1 in nucleus, and the expression of β‐catenin target genes (Figures 6B and S5B). Moreover, in DCLK1‐overexpressing cells, we observed that CCAR1 deletion by siRNA led to the reduced DCLK1‐mediated β‐catenin level in nucleus and expression of β‐catenin target genes (Figure 6C). Furthermore, we discovered that CCAR1 was binding to β‐catenin in nucleus but not in cytoplasm (Figure S6A). To further confirm that DCLK1 promoted transcriptional activation of β‐catenin, we treated DCLK1‐overexpressing cells with β‐catenin inhibitor (IWR‐1‐endo). Data showed that β‐catenin inhibitor also resulted in the decreased DCLK1‐mediated β‐catenin level in nucleus and the decreased expression of β‐catenin target genes (Figure 6D). Collectively, these results demonstrated that DCLK1 positively regulated β‐catenin signalling via CCAR1.

### 2.7 DCLK1 promotes 5‐fluorouracil resistance by CCAR1/β‐catenin signalling‐mediated cancer stemness

**FIGURE 7:**
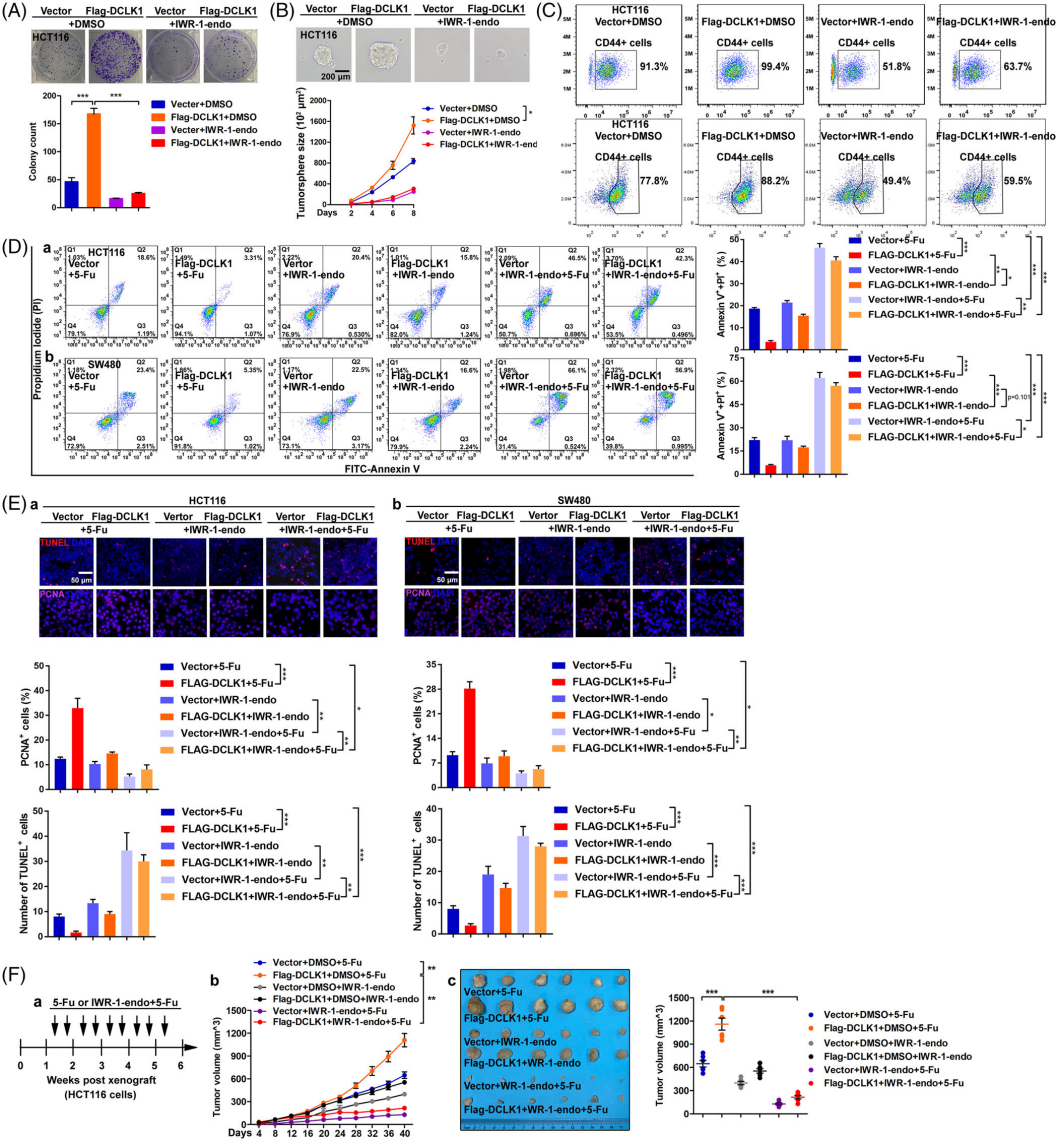
Doublecortin‐like kinase 1 (DCLK1) promotes 5‐fluorouracil resistance by cell cycle and apoptosis regulator 1 (CCAR1)/β‐catenin signalling‐mediated cancer stemness. (A) Colony formation ability of HCT116 cells transfected with the indicated constructs for 48 h and treated with IWR‐1‐endo for 24 h, was determined by clonogenic assay (*n* = 3). (B) Self‐renewal activity of HCT116 cells transfected with the indicated constructs for 48 h and treated with IWR‐1‐endo for 24 h, was assessed by tumoursphere‐forming assay (*n* = 3). Scale bar represents 200 μm. (C) CD44+ cells and CD133+ cells of HCT116 cells transfected with the indicated constructs for 48 h and treated with/without IWR‐1‐endo for 24 h, was analysed by FACS assay. (D) Apoptosis of HCT116 and SW480 cells. Cells were transfected with the indicated constructs for 48 h, and treated with 5‐fluorouracil or 5‐fluorouracil + IWR‐1‐endo for 24 h, then subjected to FITC‐annexin V/propidium iodide staining and analysed by FACS (*n* = 3). (E) TUNEL staining (red) and PCNA staining (purple) in HCT116 and SW480 cells. The cells were transfected with the indicated constructs for 48 h, and treated with 5‐fluorouracil or 5‐fluorouracil + IWR‐1‐endo for 24 h, then subjected to immunofluorescence staining. (F) HCT116 stable cell lines transfected with the indicated constructs were subcutaneously injected to nude mice, respectively (*n* = 6), then treated with 5‐fluorouracil or 5‐fluorouracil + IWR‐1‐endo twice a week for 6 weeks (a). The volumes of subcutaneous xenograft tumour were observed (b–d). Data are expressed as mean ± SD. *p < .05, **p < .01 and ***p < .001

It was known that the Wnt/β‐catenin pathway was important for cell proliferation and self‐renewal of CSCs.^46^, ^47^ Considering that our results had proved that DCLK1 promoted cancer stemness, and DCLK1 positively regulated β‐catenin signalling via CCAR1, we speculated that DCLK1 maintained cancer stemness through β‐catenin signalling. Indeed, we observed that, when treated with β‐catenin inhibitor (IWR‐1‐endo), DCLK1‐mediated proliferative potential and self‐renewal ability of DCLK1‐overexpressing cells were attenuated (Figures 7A,B and S7A–D), and DCLK1‐mediated CD44+ and CD133+ cells in DCLK1‐overexpressing cells was decreased (Figures 7C and S7E). These results suggested that the blocked β‐catenin inhibited DCLK1‐mediated cancer stemness.

Then we investigated the effect of inhibition of DCLK1‐mediated cancer stemness by blocking β‐catenin on DCLK1‐mediated 5‐fluorouracil resistance. Data showed that, when treated with β‐catenin inhibitor, the apoptosis (Figure 7D) and the TUNEL staining of DCLK1‐overexpressing cells were dramatically increased, while the PCNA staining was decreased (Figure 7E). These data indicated that DCLK1‐mediated 5‐fluorouracil resistance in CRC cells was attenuated by β‐catenin inhibitor. Moreover, in subcutaneous xenograft tumour models treated with 5‐fluorouracil or/and β‐catenin inhibitor, we found that the tumour volumes of DCLK1‐overexpressing group were substantially decreased (Figure 7F). It supported that blocking β‐catenin sensitised DCLK1‐overexpressing CRC cells to 5‐fluorouracil in vivo. Collectively, these data suggested that DCLK1 promoted 5‐fluorouracil resistance by β‐catenin signalling‐mediated cancer stemness.

### 2.8 Targeting DCLK1 suppresses 5‐fluorouracil resistance in CRC cells

**FIGURE 8:**
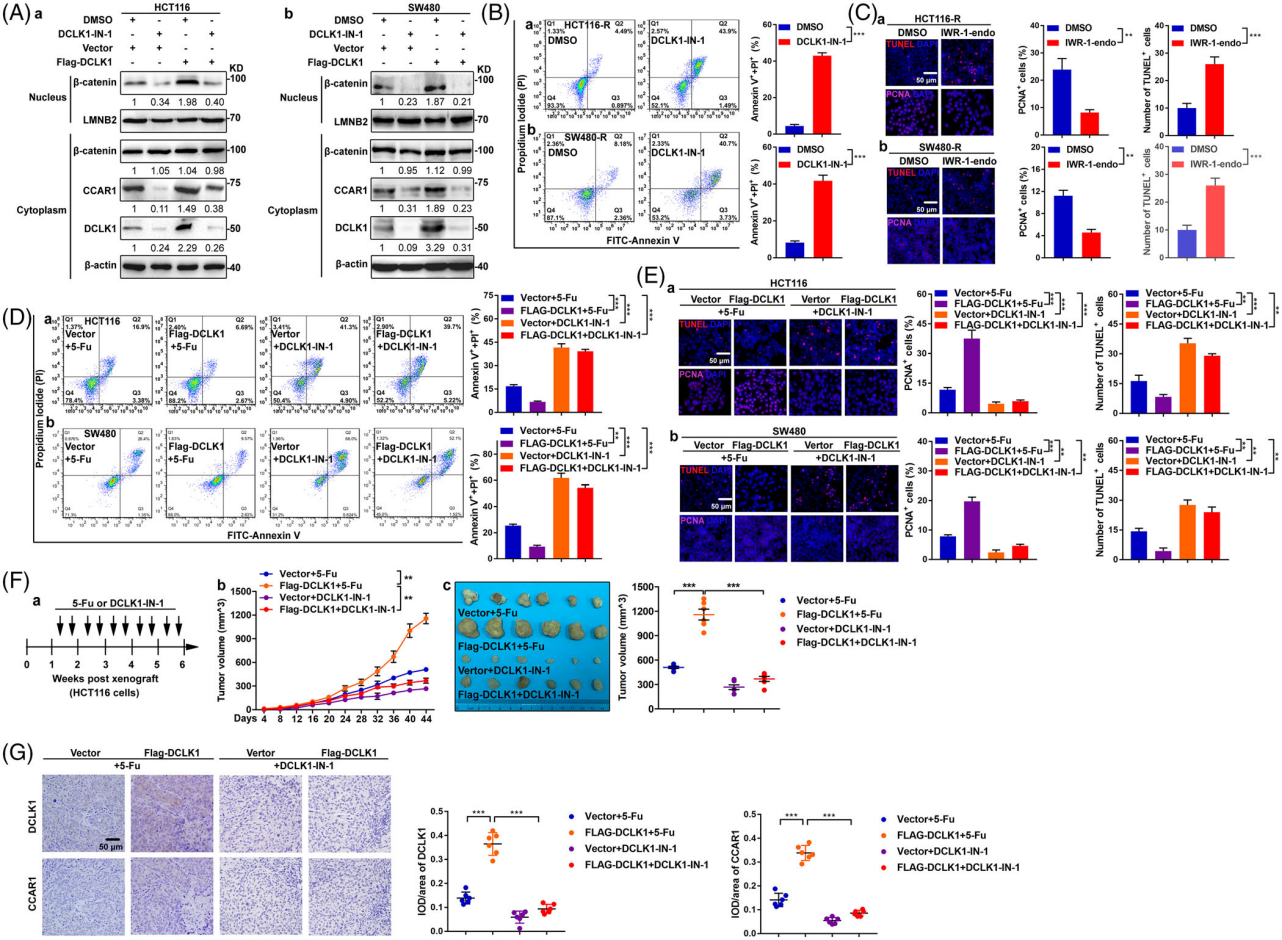
Targeting doublecortin‐like kinase 1 (DCLK1) suppresses 5‐fluorouracil resistant colorectal cancer (CRC) cells. (A) HCT116 and SW480 cells were transfected with the indicated constructs for 48 h and treated with/without DCLK1 inhibitor (DCLK1‐IN‐1) for 24 h. Protein in nucleus or cytoplasm was collected. Western blotting was conducted to detect the indicated protein level. (B) Apoptosis of HCT116‐R and SW480‐R cells. Cells were treated with DCLK1‐IN‐1 for 24 h, then subjected to FITC‐annexin V/propidium iodide staining and analysed by FACS (*n* = 3). (C) TUNEL staining (red) and PCNA staining (purple) in HCT116‐R and SW480‐R cells. Cells were treated with DCLK1‐IN‐1 for 24 h, then subjected to immunofluorescence staining. (D) Apoptosis of HCT116 and SW480 cells. Cells were transfected with the indicated constructs for 48 h, and treated with 5‐fluorouracil or DCLK1‐IN‐1 for 24 h, then subjected to FITC‐annexin V/propidium iodide staining and analysed by FACS (*n* = 3). (E) TUNEL staining (red) and PCNA staining (purple) in HCT116 and SW480 cells. Cells were transfected with the indicated constructs for 48 h, and treated with 5‐fluorouracil or DCLK1‐IN‐1 for 24 h, then subjected to immunofluorescence staining. (F) HCT116 stable cell lines transfected with the indicated constructs were subcutaneously injected to nude mice, respectively (*n* = 6), then treated with 5‐fluorouracil or DCLK1‐IN‐1 twice a week for 5 weeks (a). The volumes of subcutaneous xenograft tumour were observed (b–d). (G) Representative immunohistochemical staining of DCLK1 and cell cycle and apoptosis regulator 1 (CCAR1) in subcutaneous xenograft tumours. Data are expressed as mean ± SD. *p < .05, **p < .01 and ***p < .001

Having proved that DCLK1 promoted 5‐fluorouracil resistance through CCAR1/β‐catenin pathway‐mediated cancer stemness, we wondered whether targeting DCLK1 could suppress 5‐fluorouracil resistant CRC cells. We first assessed the effect of DCLK1 inhibitor (DCLK1‐IN‐1, a selective inhibitor of DCLK1)^48^ on DCLK1‐mediated CCAR1/β‐catenin pathway and cancer stemness. It was found that, when treated with DCLK1 inhibitor, the elevated levels of β‐catenin in nucleus, CCAR1 and DCLK1 in DCLK1‐overexpressing CRC cells were dramatically attenuated (Figure 8A). And DCLK1‐mediated proliferative potential and self‐renewal ability of DCLK1‐overexpressing cells were greatly impaired, and DCLK1‐mediated CD44+ and CD133+ cells in DCLK1‐overexpressing cells was decreased by treating with DCLK1 inhibitor (Figure S8A–D). Moreover, we proved that the decreased level of β‐catenin in nucleus resulted by DCLK1 inhibitor was rescued by exogenous expression β‐catenin (Figure S9A). Meanwhile, the proliferative potential, the self‐renewal ability, and CD44+ and CD133+ cells of primary CRC cells impaired by DCLK1 inhibitor, were rescued by exogenous expression β‐catenin (Figure S9B–D). These results demonstrated that targeting DCLK1 suppressed CCAR1/β‐catenin pathway‐mediated cancer stemness.

**FIGURE 9:**
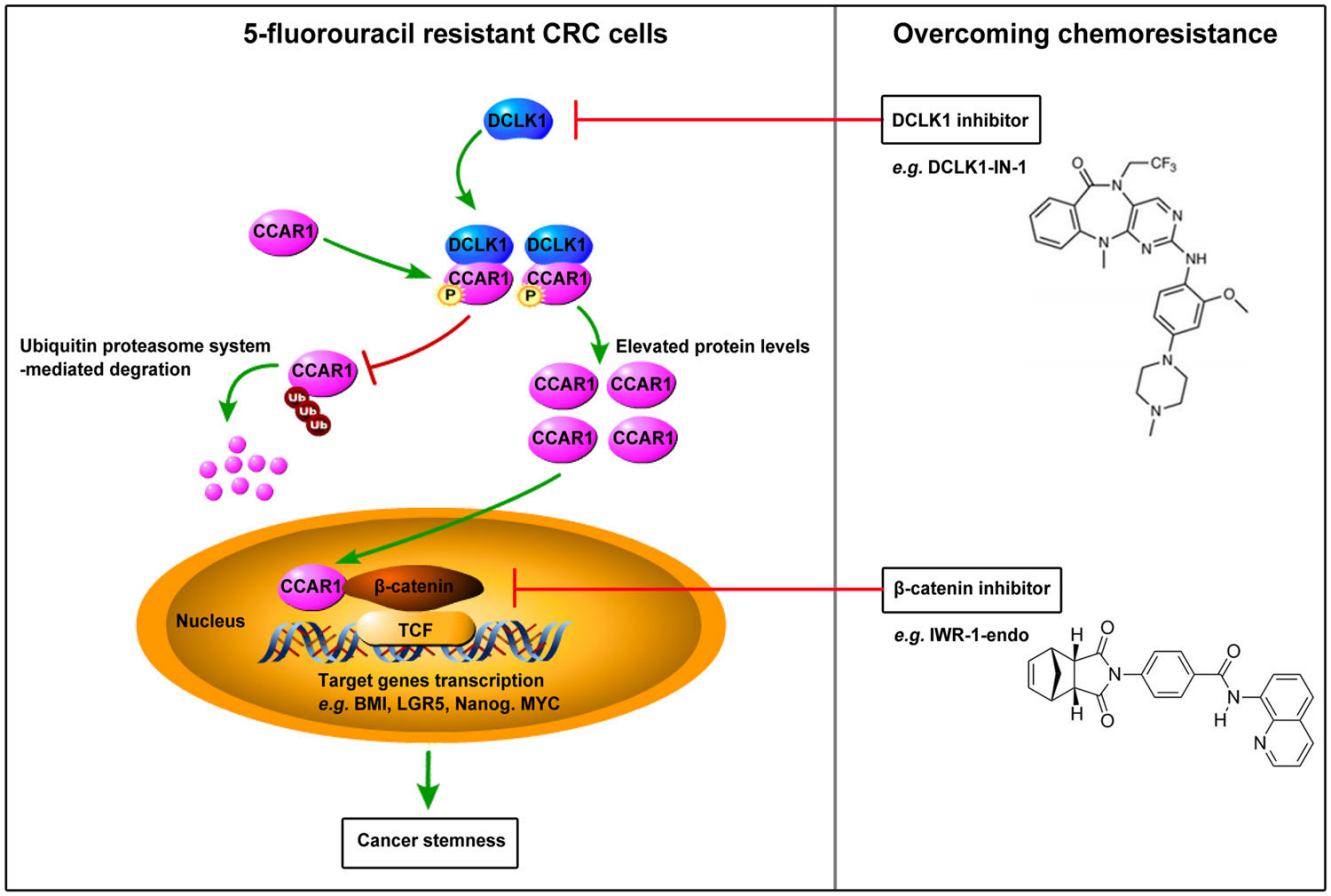
A proposed model shows the mechanism of doublecortin‐like kinase 1 (DCLK1)‐promoting 5‐fluorouracil resistance in colorectal cancer (CRC) through cell cycle and apoptosis regulator 1 (CCAR1)/β‐catenin pathway‐mediated cancer stemness. High level of DCLK1 leads to its binding to CCAR1 and phosphorylates CCAR1 at Ser343 site, which is essential for CCAR1 stabilisation. And subsequently it results in transcriptional activation of β‐catenin that promotes cancer stemness and 5‐fluorouracilresistance. However, DCLK1 inhibitor or β‐catenin inhibitor can block CCAR1/β‐catenin pathway‐mediated cancer stemness, which consequently suppresses 5‐fluorouracil resistant CRC cells

Next we estimated the effect of targeting DCLK1 on 5‐fluorouracil resistant CRC cells. Interestingly, we found that, when treated with DCLK1 inhibitor, the apoptosis (Figure 8B) and the TUNEL staining of 5‐fluorouracil resistant CRC cells were significantly increased, while the PCNA staining was decreased (Figure 8C). It indicated that DCLK1 inhibitor suppressed 5‐fluorouracil resistant CRC cells. Moreover, we investigated the influence of DCLK1 inhibitor in DCLK1‐mediated 5‐fluorouracil resistance. Data showed that, when treated with DCLK1 inhibitor, the apoptosis (Figure 8D) and the TUNEL staining of DCLK1‐overexpressing cells were dramatically increased, while the PCNA staining was decreased (Figure 8E). However, the 5‐fluorouracil sensitivity of CRC cells induced by DCLK1 inhibitor was impaired by exogenous expression β‐catenin (Figure S9E). These results showed that DCLK1‐mediated 5‐fluorouracil resistance in CRC cells was attenuated by DCLK1 inhibitor. Furthermore, we wondered whether DCLK1 inhibitor suppressed DCLK1‐mediated 5‐fluorouracil resistant CRC cells in subcutaneous xenograft tumour models. Importantly, it was found that, when treated with DCLK1 inhibitor, the tumour volumes of DCLK1‐overexpressing group were substantially decreased (Figure 8F). In addition, IHC analysis confirmed that DCLK1 inhibitor decreased the protein expression of DCLK1 and CCAR1 in subcutaneous tumour in both of DCLK1‐overexpressing and the control groups (Figure 8G). Therefore, these results supported that DCLK1 inhibitor suppressed 5‐fluorouracil resistant CRC cells in vitro and in vivo. Collectively, these data suggested that targeting DCLK1 suppressed 5‐fluorouracil resistant CRC cells through inhibiting CCAR1/β‐catenin pathway‐mediated cancer stemness (Figure 9).

## 3 DISCUSSION

Heretofore 5‐fluorouracil‐based chemotherapy is very important for locally advanced or metastasis.^6^, ^7^ Unfortunately, chemotherapy resistance is a major obstacle for treatment success in CRC patients. It is well established that CSCs were closely related to tumour recurrence, metastasis and therapy resistance.^35^ Herein, we highlighted a mechanism linking a CSCs maker DCLK1 and 5‐fluorouracil resistance, and demonstrated that DCLK1 inhibitor suppressed 5‐fluorouracil resistant CRC cells through inhibiting CCAR1/β‐catenin pathway‐mediated cancer stemness.

Accumulating evidences showed that DCLK1 was overexpressed in several types of tumours, especially in CRC^9^, ^10^, ^11^, ^12^, ^13^, ^14^, ^15^ and pancreatic cancer.^16^, ^17^ Besides, elevated levels of DCLK1 were closely correlated with poor outcomes and tumour recurrence and metastasis.^27^ In the current research, we also found that patients with high DCLK1 had poorer outcomes. Importantly, DCLK1 expression in 5‐fluorouracil resistant patients was higher than 5‐fluorouracil sensitive patients, which indicated a correlation between DCLK1 and 5‐fluorouracil resistance in CRC. Mounting evidences suggested that DCLK1, a CSCs marker, was functionally involved in maintaining cancer stemness.^31^, ^32^, ^33^, ^34^ We also showed that DCLK1 positively regulated the proliferative potential and self‐renewal ability of CRC cells, and the proportion of CD44+ and CD133+ CRC cells. Considering that CSCs were critical for cancer initiation, progression, metastasis, relapse and therapy resistance,^35^ we found that DCLK1, a CSCs marker, weakened the sensitivity of CRC cells to 5‐fluorouracil. Collectively, we demonstrated that DCLK1 was closely correlated with 5‐fluorouracil resistance, and functionally promoted cancer stemness and 5‐fluorouracil resistance in CRC.

To further explore the underlying mechanism of DCLK1‐mediated cancer stemness and 5‐fluorouracil resistance, we identified CCAR1 as a protein interacting with DCLK1, and elucidated that the C‐terminal domain (Pro301 to Met729) of DCLK1 was indispensable for interacting with CCAR1. Moreover, we uncovered that DCLK1 positively regulated the stability of CCAR1. Although DCKL1 contains a C‐terminal kinase domain with similarity to the CaMK family, few substrate of DCLK1 had been reported. We revealed that C‐terminal catalytic domain of DCLK1 was essential for CCAR1 stabilisation, and the Ser343 site in a conserved R‐NB‐X‐S/T‐HP motif was responsible for DCLK1‐mediated CCAR1 stabilisation. As a whole, for the first time, we demonstrated that DCLK1 interacted with CCAR1 through the C‐terminal domain to positively regulate the stability of CCAR1, and DCLK1 phosphorylated CCAR1 at Ser343 site which was essential for CCAR1 stabilisation.

Although a few studies have reported the association of DCLK1 and Wnt/β‐catenin signalling,^25^, ^49^ of which the mechanism remains unclear. In our study, we clarified that DCLK1 positively regulated the protein level of β‐catenin in nucleus via CCAR1. CCAR1 is a functional binding partner of β‐catenin and assists β‐catenin in transcriptional activation of target genes.^43^ We also proved that DCLK1 positively regulated the expression of β‐catenin target genes, which could be inhibited by β‐catenin inhibitor. These data indicated that DCLK1 positively regulated β‐catenin signalling via CCAR1. It is well known that aberrant activation of Wnt/β‐catenin signalling is indispensible for maintaining CSCs.^46^, ^47^ Indeed, we observed that DCLK1 promoted cancer stemness in CRC, and β‐catenin inhibitor suppressed DCLK1‐mediated cancer stemness. Importantly, we demonstrated that DCLK1‐mediated 5‐fluorouracil resistance was attenuated by β‐catenin inhibitor. Therefore, our findings suggested that DCLK1 promoted 5‐fluorouracil resistance in CRC by CCAR1/β‐catenin pathway‐mediated cancer stemness.

It was well reported that down‐regulating the expression or inhibiting the kinase activity of DCLK1 could improve the sensitivity to chemoradiotherapy.^25^, ^39^, ^40^, ^41^ Especially, Ferguson and Nabet^48^ discovered DCLK1‐IN‐1, a selective inhibitor of DCLK1, and demonstrated the activity of DCLK1‐IN‐1 against clinically relevant patient‐derived pancreatic ductal carcinoma organoid models. In the current study, we applied DCLK1‐IN‐1 to targeting DCLK1, and found that DCLK‐IN‐1 suppressed 5‐fluorouracil resistant CRC cells by inhibiting CCAR1/β‐catenin pathway‐mediated cancer stemness. Therefore, our results provided an evidence to prove that targeting DCLK1 overcame 5‐fluorouracil resistance in CRC.

In summary, our research uncovered that DCLK1 was correlated with 5‐fluorouracil resistance, and functionally promoted cancer stemness and 5‐fluorouracil resistance in CRC. Mechanistically, we elucidated that DCLK1 promoted 5‐fluorouracil resistance in CRC by CCAR1/β‐catenin pathway‐mediated cancer stemness. Importantly, we demonstrated that DCLK1 inhibitor could block CCAR1/β‐catenin pathway‐mediated cancer stemness and consequently suppressed 5‐fluorouracil resistant CRC cells in vitro and in vivo. Therefore, this study identified DCLK1 as a promising target for eliminating CSCs and overcoming 5‐fluorouracil resistance in CRC.

## 4 MATERIALS AND METHODS

### 4.1 Cell cultures and reagents

Human CRC cell lines (HCT116 and SW480) and 293T cells were purchased from Cell Bank, Type Culture Collection, Chinese Academy of Sciences (CBTCCCAS, Shanghai, China). And 5‐fluorouracil resistant cell lines (HCT116‐R and SW480‐R) were established by a previously described method by treating the respective parental cells with increasing concentrations of 5‐fluorouracil over 8 months.^50^ Flag‐DLK1and shDCLK1 stable cell lines were established using lentivirus transfection and resistance selection. All cell lines were cultured in Dulbecco's modified Eagle's medium or Roswell Park Memorial Institute 1640 (GIBCO, USA) supplemented with 10% fetal bovine serum (GIBCO), 100 U/ml penicillin and 100 μg/ml streptomycin, and were maintained in a humidified incubator adjusted with 5% CO_2_ at 37°C. All cell lines used in our research were authenticated by Short Tandem Repeat profiling and tested for mycoplasma contamination by CBTCCCAS.

### 4.2 Reagents and antibodies

Some reagents were used in this study. GV248‐shRNA plasmid, GV141‐DCLK1 plasmid, GV219‐CCAR1 plasmid and siRNA were designed and constructed by Genechem Technologies (Shanghai, China). The sequences of shRNA were shDCL1‐1: 5′‐TTCCATGACAAGATACAGTTC‐3′, shDCL1‐2: 5′‐TATCGTTCTGTTATTGTAGCT‐3′. The sequences of siRNA were siCCAR1 #1: 5′‐CCAGCAAACUAUCAGUUAAdTdT‐3′ (sense), 5′‐UUAACUGAUAGUUUGCUGGdAdG‐3′ (antisense); siCCAR1 #2: 5′‐GCUGGGAAAUUACUGCAAUdTdT‐3′ (sense), 5′‐AUUGCAGUAAUUUCCCAGCdTdG‐3′ (antisense); siCCAR1 #3: 5′‐CCAACAUCCUGCUAGACUUdTdT‐3′ (sense), 5′‐AAGUCUAGCAGGAUGUUGGdAdA‐3′ (antisense). Lipofectamine 2000 was purchased from Invitrogen, USA. And several primary and secondary antibodies were used in this study. Anti‐DCLK1 antibody (Proteintech, 21699‐1‐AP; 1:1000 dilution), anti‐β‐actin antibody (Proteintech, 66009‐1‐lg; 1:1000 dilution), anti‐β‐catenin antibody (Proteintech, 66379‐1‐lg; 1:2000 dilution), anti‐LMNB2 (Proteintech, 10895‐1‐AP; 1:1000 dilution), anti‐HA (Proteintech, 66006‐s‐lg; 1:1000 dilution), anti‐MYC (Proteintech, 60003‐2‐lg; 1:1000 dilution) and anti‐Flag (Proteintech, 66008‐3‐lg; 1:1000 dilution) were purchased from Proteintech, USA. Horseradish peroxidase (HRP)‐conjugated goat anti‐rabbit immunoglobin G (IgG) (H + L) secondary antibody (Cell Signaling, 14708; 1:3000 dilution) and HRP‐conjugated goat anti‐mouse IgG (H + L) secondary antibody (Cell Signaling, 14709; 1:3000 dilution) were purchased from Cell Signaling, USA. Anti‐CCAR1 antibody (BIOGOT, BS8140; 1:1000 dilution) was purchased from BIOGOT, USA. Protein A‐agarose was purchased from Santa Cruz Biotechnology, USA. And 5‐fluorouracil was purchased from Sigma, USA. IWR‐1‐endo and DCLK1‐IN‐1 were purchased from Selleck, USA.

### 4.3 Proteins extraction, immunoprecipitation, mass spectrometry and Western blotting

Cytoplastic cell lysates were prepared by NP‐40 lysis buffer (Meilunbio, China) for immunoprecipitation experiments and RIPA lysis buffer (Beyotime, China) with inhibitors for other proteins. Nuclear proteins were isolated by a kit according to the modified protocols from the manufacturer (Thermo, USA). For immunoprecipitation experiments, the cell lysates and antibodies were incubated overnight with protein A/G agarose (Santa Cruz Biotechnology) at 4°C, and washed with NP‐40 buffer five times followed by MS analysis or Western blotting. MS analysed by Q Exactive mass spectrometer (Thermo), which was supported by Genechem Technologies. Cell lysates were electrophoresed and transferred to nitrocellulose membrane (Millipore, USA), blocked in 5% non‐fat milk, then incubated with primary antibodies and HRP‐conjugated secondary antibodies, and visualised through chemiluminescence system (Bio‐Rad, USA). All experiments were repeated for at least three times.

### 4.4 mRNA sequencing and real‐time quantitative PCR

Total RNA was extracted from cells (5‐fluorouracil resistant cell lines and parental cell lines) by using Total RNA Kit (Omega, China). For mRNA sequencing, total RNA was performed to purify and fragment mRNA, and synthesise double‐strand cDNA. Then PCR amplification and mRNA sequencing were conducted in Illumina Hiseq 2500 (Illumina, USA). For real‐time quantitative PCR, a cDNA Reverse Transcription Kit (Takara, Japan) was used to synthesise cDNAs. Then SYBR Green PCR Master Mix (Invitrogen) was taken to perform real‐time quantitative PCR experiments in Applied Biosystems 7500 Instrument (Thermo). The relative mRNA levels of target genes were calculated by the ΔΔCT method. The sequences of primers used in the study are shown in Table S1.

### 4.5 Apoptosis assay

Cells were transfected with the indicated plasmids, then treated with 5‐fluorouracil (2 μg/ml, Sigma), IWR‐1‐endo (80 ng/ml, Selleck) or DCLK1‐IN‐1 (100 ng/ml, Selleck). After 48 h, cells were harvested and washed with PBS, and then stained with annexin V‐FITC (Fluorescein Isothiocyanate) and propidium iodide (Thermo). The samples were analysed by flow cytometry (Thermo). All experiments were repeated independently for three times.

### 4.6 Animal studies

All animal experiments were approved by the Medical Ethics Committee of Union Hospital, Tongji Medical College, Huazhong University of Science and Technology. BALB/c nude mice (4–6 weeks old) were used for animal studies. HCT116 cells, HCT116‐R cells, HCT116‐sh control and ‐shDCLK1 stable cells, or HCT116‐vector and ‐Flag‐DCLK1 stable cells (1 × 10^6^) were subcutaneously injected into mice (*n* = 6). One week after tumour cell inoculation, mice in the indicated groups were administered with 5‐fluorouracil (40 mg/kg), IWR‐1‐endo (160 μg/kg) or DCLK1‐IN‐1 (200 μg/kg) twice a week for 3 or 5 weeks. The tumour size was measured per 3 days, and the tumour volume was calculated by the formula, volume = (longitudinal × transverse^2^)/2.

### 4.7 Immunohistochemistry staining

Paraffin‐embedded CRC specimens were collected from our hospital (*n* = 96). The research was approved by the institutional Medical Ethics Committee of Union Hospital, Tongji Medical College, Huazhong University of Science and Technology. Informed consents were obtained from all involved patients. The anti‐DCLK1 and ‐CCAR1 antibodies were used for the primary reaction, followed by immunoperoxidase staining. Positive cells were visualised using a 3,3′‐diaminobenzidine substrate and the sections were counterstained with haematoxylin. Mean densities of IHC staining were determined by Image‐J. IHC results were confirmed by two pathologists.

### 4.8 Immunofluorescence staining

HCT116 xenograft tumour tissues were formalin‐fixed and paraffin‐embedded, then tissue slides were treated dewaxed and rehydrated slices to recover antigen using sodium citrate buffer. Cells with the indicated treatment were seeded on poly‐L‐lysine and collagen I‐coated cover glasses were fixed with 4% paraformaldehyde and preimmobilised with Triton X‐100 0.3%. After incubation with 10% normal goat serum for 1 h, the slices were incubated with anti‐TUNEL antibody or anti‐PCNA antibody (Abcam, UK) at 4°C overnight. Then the slices were washed and incubated with Alexa Fluor 647 conjugated goat anti‐rabbit IgG (Abcam) for 1 h. All nuclei were counterstained with 4′,6‐diamidino‐2‐phenylindole.

### 4.9 Cell proliferation assay

Cells transfected with the indicated plasmids were treated with/without IWR‐1‐endo (80 ng/ml, Selleck) or DCLK1‐IN‐1 (100 ng/ml, Selleck). After the indicated times, CCK‐8 solution (10 μl, Dojindo, Japan) was added and incubated for 2 h. The absorbance values at 450 nm were measured using a microplate reader (Thermo). All experiments were repeated independently for three times.

### 4.10 Clonogenic assay

Cells transfected with the indicated plasmids were treated with/without IWR‐1‐endo (80 ng/ml, Selleck) or DCLK1‐IN‐1 (100 ng/ml, Selleck) and cultured for 14 days. After fixation by methanol and staining by crystal violet, the colonies containing more than 50 cells were calculated under the optical microscope.

### 4.11 Tumoursphere formation assay

Cells transfected with the indicated plasmids were resuspended in conditioned medium (a suitable ratio of culture medium to matrix gel) containing growth factors (B27, 20 ng/ml hEGF, 20 ng/ml basic hFGF and 4 mg/ml heparin), and seeded into poly‐HEMA‐coated six‐well plates, then treated with/without IWR‐1‐endo (80 ng/ml, Selleck) or DCLK1‐IN‐1 (100 ng/ml, Selleck). The size of individual spheres from each replicate well (*n* ≥ 3 wells) was measured under the optical microscope at 10× magnification using the Image‐J.

### 4.12 Flow cytometry

Cells were transfected with the indicated plasmids, then treated with 5‐fluorouracil, IWR‐1‐endo or DCLK1‐IN‐1. After 48 h, cells were harvested and washed with PBS, and then incubated with Phycoerythrin‐conjugated CD44 antibody (BD, USA). Fluorescence activated cell sorter analysis was performed using BD AccuriTM C6 (BD), and data were analysed using FlowJo software.

### 4.13 Statistical analysis

The statistical differences between groups were analysed by Student's *t*‐test or one‐way ANOVA with Dunnett's *T* test. Data were indicated as mean ± standard error. The correlation between DCLK1 and CCAR1 expression was analysed by linear regression. Survival of patients was estimated by the Kaplan–Meier method and log‐rank test. Statistically analysis was performed using GraphPad Prism 9. All experiments were repeated at least three times. All statistical tests were two‐sided, and *p*‐values ≤.05 were considered statistically significant.

## CONFLICT OF INTEREST

The authors declare that they have no conflict of interest.

## Supporting information

Supporting Information

Supporting Information
