# Supplementary material for: Targeting DCLK1 overcomes 5‐fluorouracil resistance in colorectal cancer through inhibiting CCAR1/β‐catenin pathway‐mediated cancer stemness
Source: Clin Transl Med. 2022 May 6;12(5):e743. doi: 10.1002/ctm2.743 (PMC9076011; doi:10.1002/ctm2.743)
Supplement: Supplementary file 1 — Supporting Information [file CTM2-12-e743-s002.pdf]

Supplementary figures S1-S9

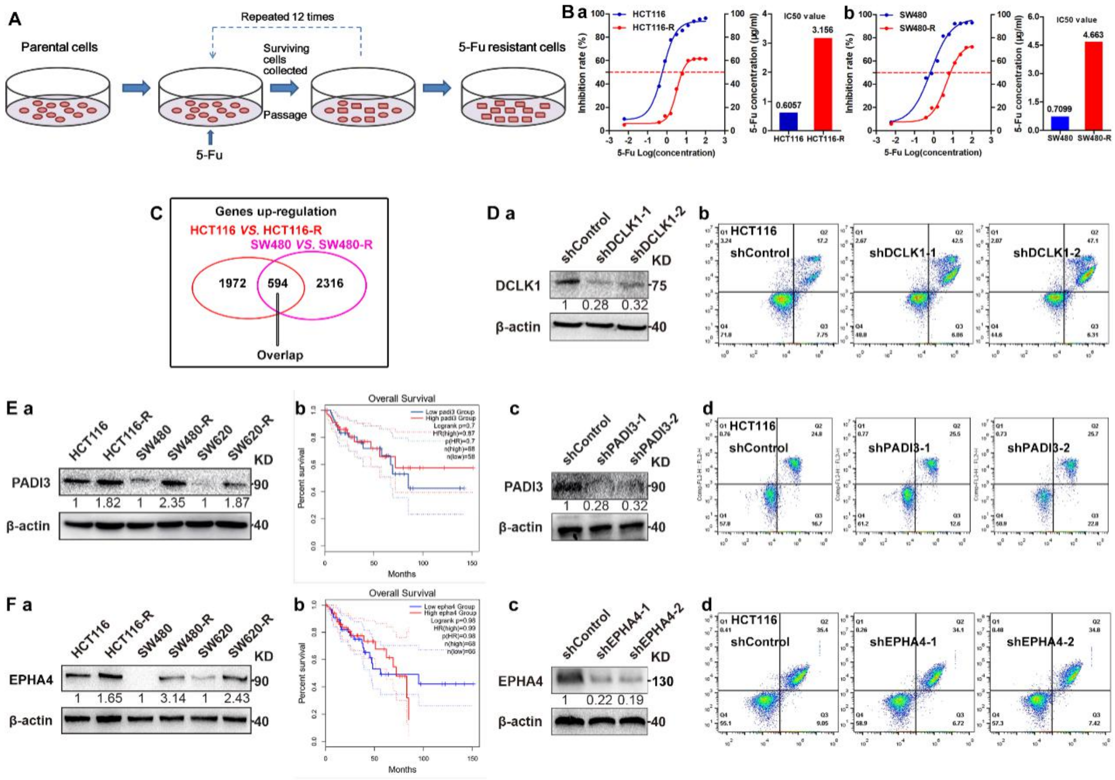

Figure S1. DCLK1 is correlated with 5-fluorouracil resistance in CRC. A. Schematic diagram for the establishment of 5-fluorouracil (5-Fu) resistant cell lines. B. The sensitivity (IC50) of 5-fluorouracil resistant cell lines or parental cell lines to 5-fluorouracil. Cells were treated with increasing doses of 5-fluorouracil for 48h and detected by CCK-8. C. D. Western blotting showed the knockdown efficiency of DCLK1 by two different shRNAs in HCT116 cells (a). Apoptosis of HCT116 cells transfected with the indicated shRNAs for 48h and treated with 5-fluorouracil for 24h, then were subjected to FITC-annexin V/propidium iodide staining and analyzed by FACS (n=3) (b). E. Western blotting showed the protein expression of three groups of Parental and drug-resistant cells (HCT116, SW480 and sw620) (a). PADI3 survival analysis data was gained from the GEPIA2 database (b). (http://gepia2.cancer-

pku.cn/#survival) Western blotting showed the knockdown efficiency of PADI3 by two different shRNAs in HCT116 cells (c). Apoptosis of HCT116 cells transfected with the indicated shRNAs for 48h and treated with 5-fluorouracil for 24h, then were subjected to FITC-annexin V/propidium iodide staining and analyzed by FACS (n=3) (d). F. Western blotting showed the protein expression of three groups of Parental and drug-resistant cells (HCT116, SW480 and sw620) (a). EPHA4 survival analysis data was gained from the GEPIA2 database (b). (http://gepia2.cancer-pku.cn/#survival) Western blotting showed the knockdown efficiency of EPHA4 by two different shRNAs in HCT116 cells (c). Apoptosis of HCT116 cells transfected with the indicated shRNAs for 48h and treated with 5-fluorouracil for 24h, then were subjected to FITC-annexin V/propidium iodide staining and analyzed by FACS (n=3) (d). Data are expressed as mean ± SD. (*$P < 0.05$, ** $P < 0.01$, and *** $P < 0.001$.)

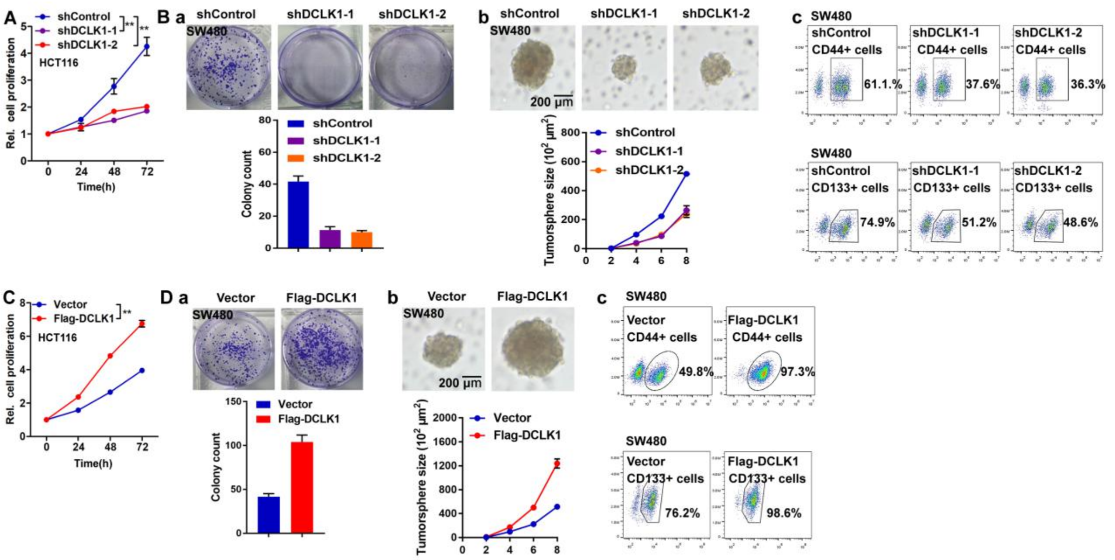

Figure S2. DCLK1 promotes cancer stemness and 5-fluorouracil resistance in CRC. A. Cell proliferation of HCT116 cells transfected with the indicated shRNAs was tested by CCK-8 assay (n=3). B. Colony formation ability and self-renewal activity of SW480 cells transfected with the indicated shRNAs was determined by clonogenic assay (a)and tumorsphere-forming assay respectively (b) (n=3).Scale bar represents 200 μm. The expression of CD44 and CD133 was detected by FACS assay. (c) C.Cell proliferation of HCT116 cells transfected with the indicated constructs was tested by CCK-8 assay (n=3). D. Colony formation ability and self-renewal activity of SW480 cells transfected with the indicated constructs was determined by clonogenic assay (a)and tumorsphere-forming assay respectively (b) (n=3).Scale bar represents 200 μm. The expression of CD44 and CD133 was detected by FACS assay. Data are expressed as mean ± SD. (**P*< 0.05, ** *P*< 0.01, and *** *P*< 0.001.)

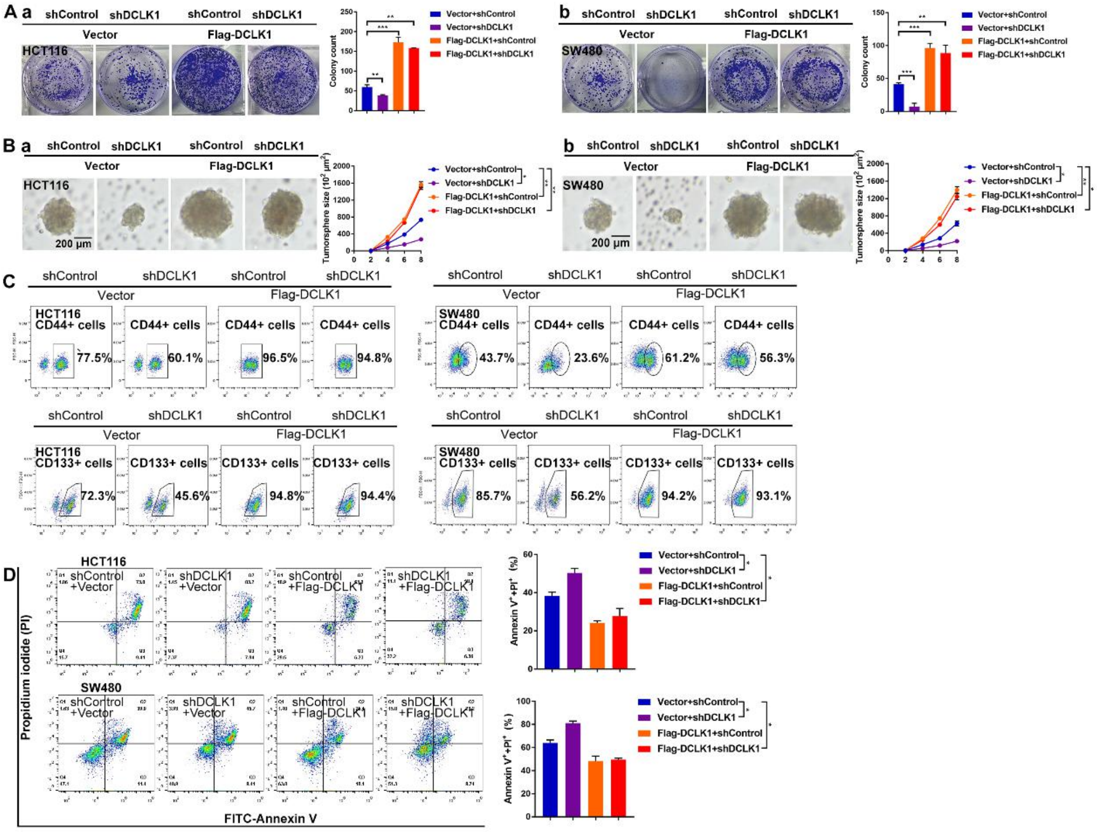

Figure S3. DCLK1 promotes cancer stemness and 5-fluorouracil resistance in CRC. A. Colony formation ability of HCT116 and SW480 cells transfected with the indicated constructs for 48h or/and treated with shDCLK1-1 for 48h, was determined by clonogenic assay (n=3). B. Self-renewal activity of HCT116 and SW480 cells transfected with the indicated constructs for 48h or/and treated with shDCLK1-1 for 48h, was assessed by tumorsphere-forming assay (n=3). Scale bar represents 200 μm. C. CD44+ cells and CD133+ in HCT116 cells transfected with the indicated constructs for 48h or/and treated with shDCLK1-1 for 48h, was analysed by FACS assay. D. Apoptosis of HCT116 and SW480 cells. Cells were transfected with the indicated constructs for 48h or/and treated with shDCLK1-1 for 48h, then subjected to FITC-Annexin V/propidium iodide staining and analyzed by FACS (n=3). Data are expressed as mean ± SD. (*P< 0.05, ** P< 0.01, and *** P< 0.001.)

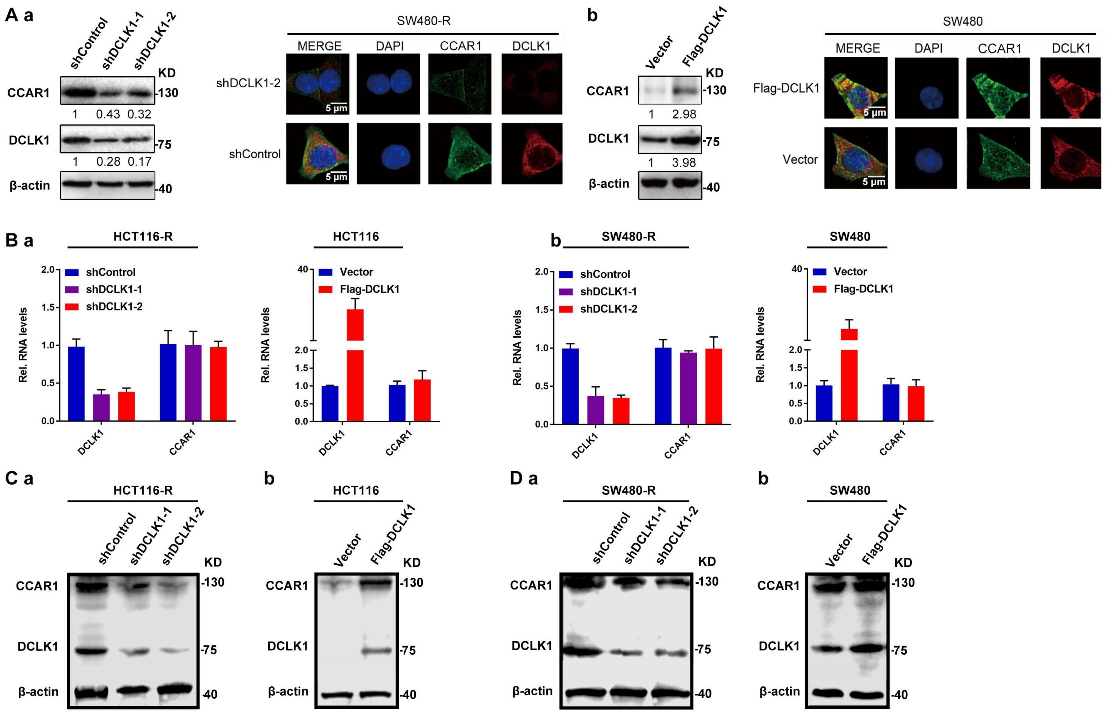

Figure S4. DCLK1 positively regulates the stability of CCAR1. A-B. The protein and mRNA levels of DCLK1 and CCAR1. CRC cells were transfected with the indicated shRNAs or constructs for 48h, and cell lysates or total RNA were collected. Western blotting and Immunofluorescence staining were conducted to detect the protein levels of DCLK and CCAR1. Real-time quantitative PCR was used to analyze the mRNA levels of DCLK and CCAR1. C. western blotting was performed to detect the protein levels of DCLK and CCAR1 on the same SDS-PAGE gel, and the whole images were provided for some key points.

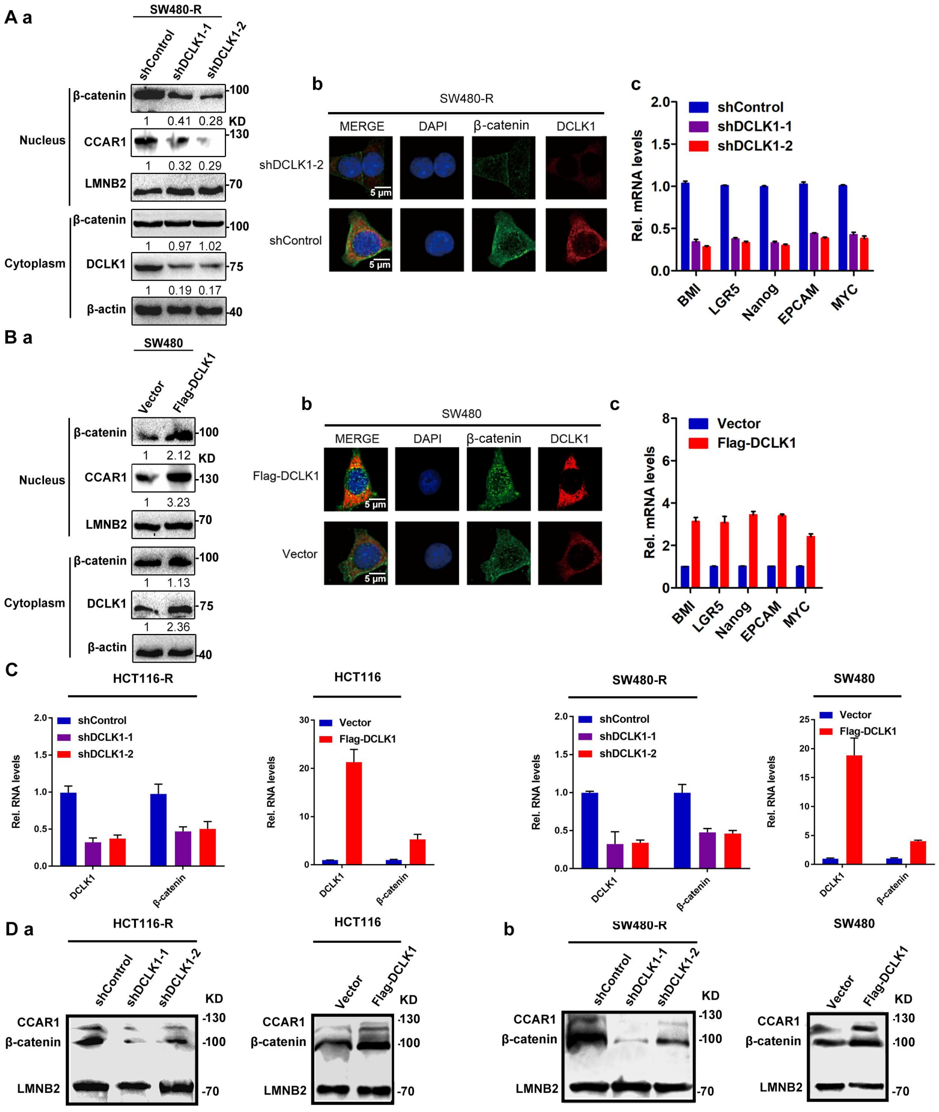

Figure S5. DCLK1 positively regulates β-catenin signaling via CCAR1. A. SW480-R cells were transfected with the indicated shRNAs for 48h. Protein in nucleus or cytoplasm, and total RNA were collected. Western blotting was conducted to detect the indicated protein level, and RT-qPCR was performed to analyse the expression of β-catenin target genes (n=3). Immunofluorescence staining was used to detect the expression of DCLK (red) and β-catenin (green). B. SW480 cells were transfected with the indicated constructs for 48h. Protein in nucleus or cytoplasm, and total mRNA were collected. Western blotting was conducted to detect the indicated protein level, and RT-qPCR was performed to analyse the expression of β-catenin

target genes (n=3). Immunofluorescence staining was used to detect the expression of DCLK (red) and β-catenin (green). C. CRC cells were transfected with the indicated shRNAs or constructs for 48h, and total RNA were collected. Real-time quantitative PCR was used to analyze the mRNA levels of DCLK and β-catenin. C. western blotting was performed to detect the protein levels of DCLK and β-catenin on the same SDS-PAGE gel, and the whole images were provided for some key points.

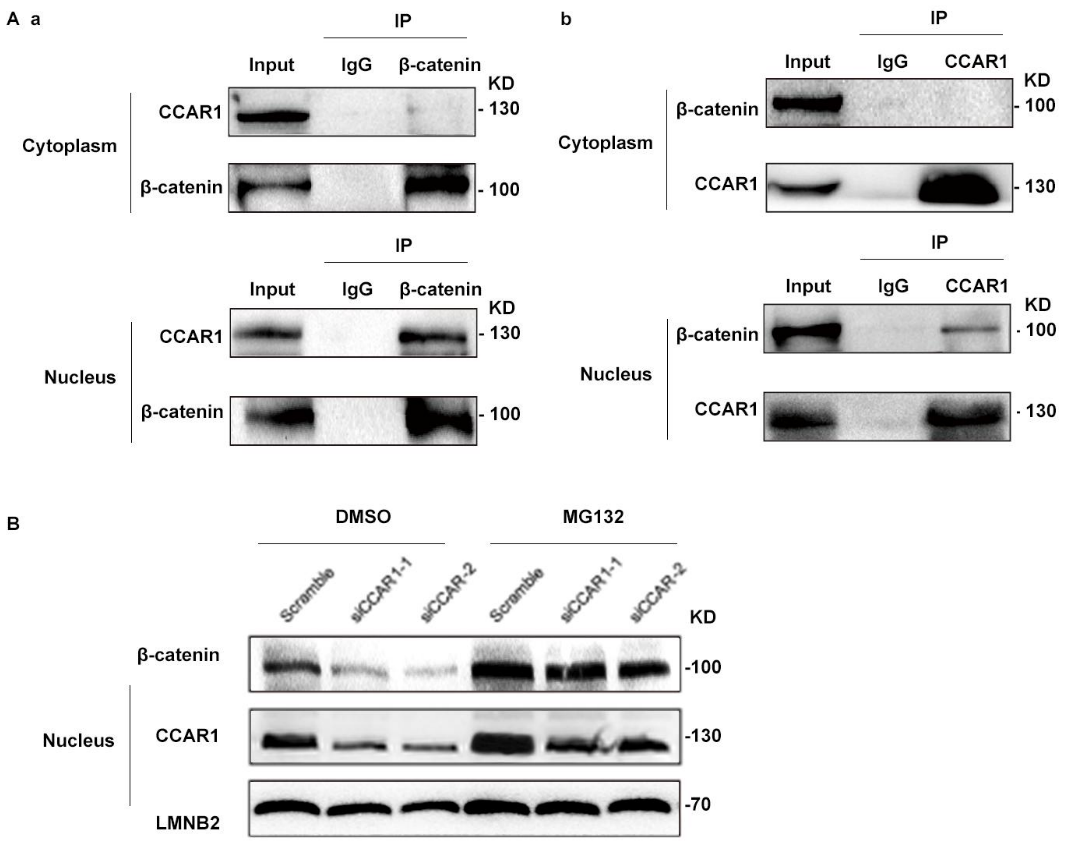

Figure S6. CCAR1 interacts with β-catenin to positively regulate β-catenin in nucleus. A. The nuclear lysates of HCT116 cells were collected and analysed by immunoprecipitation (IP) using S-protein agarose beads and western blotting with the indicated antibodies (n=3). B. HCT116 cells were transfected with siCCAR1, and then treated with MG132 (proteasome inhibitor). Then the nuclear lysates of HCT116 cells were collected and analysed by western blotting with the indicated antibodies (n=3).

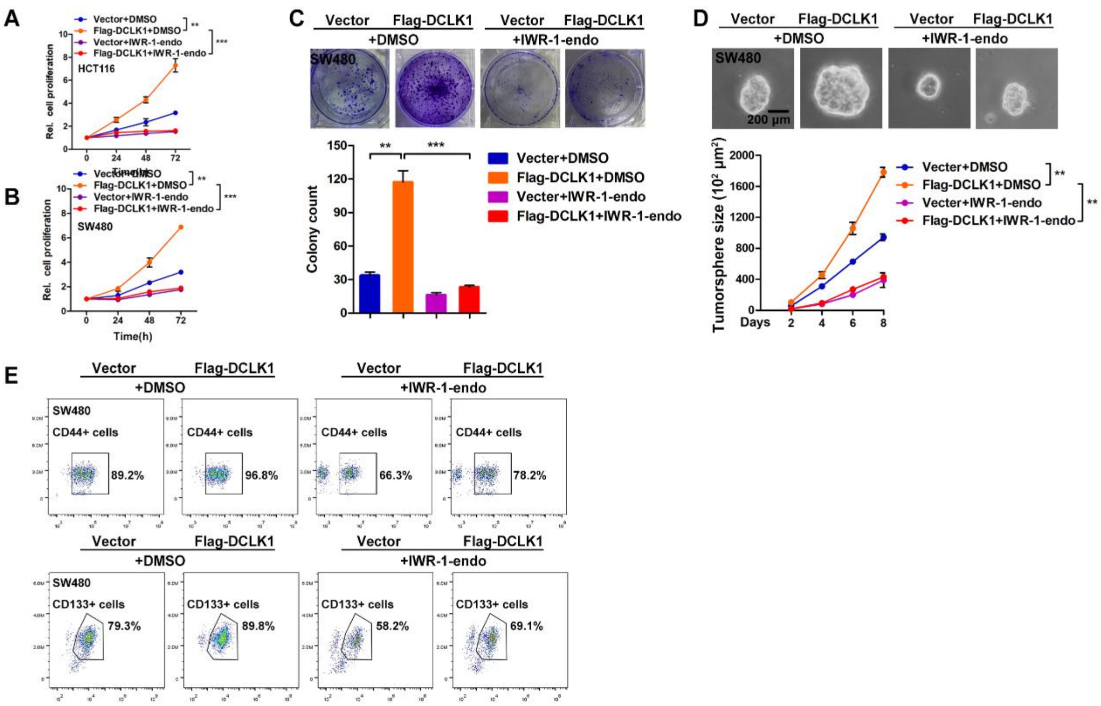

Figure S7. DCLK1 promotes 5-fluorouracil resistance by CCAR1/β-catenin signaling-mediated cancer stemness. A. Cell proliferation of HCT116 cells transfected with the indicated constructs or/and IWR-1-endo was tested by CCK-8 assay (n=3). B. Cell proliferation of HCT116 cells transfected with the indicated constructs or/and IWR-1-endo was tested by CCK-8 assay (n=3). C. Colony formation ability of SW480 cells transfected with the indicated constructs for 48h and treated with IWR-1-endo for 24h, was determined by clonogenic assay (n=3). D. Self-renewal activity of SW480 cells transfected with the indicated constructs for 48h and treated with IWR-1-endo for 24h, was assessed by tumorsphere-forming assay (n=3). Scale bar represents 200 μm. D. CD44+ cells and CD133+ in SW480 cells transfected with the indicated constructs for 48h and treated with/without IWR-1-endo for 24h, was analysed by FACS assay.E.CD44+ cells and CD133+ in SW480 cells transfected with the indicated constructs for 48h and treated with/withoutIWR-1-endo for 24h,

was analysed by FACS assay. Data are expressed as mean ± SD. (**P*< 0.05, ** *P*< 0.01, and *** *P*< 0.001.)

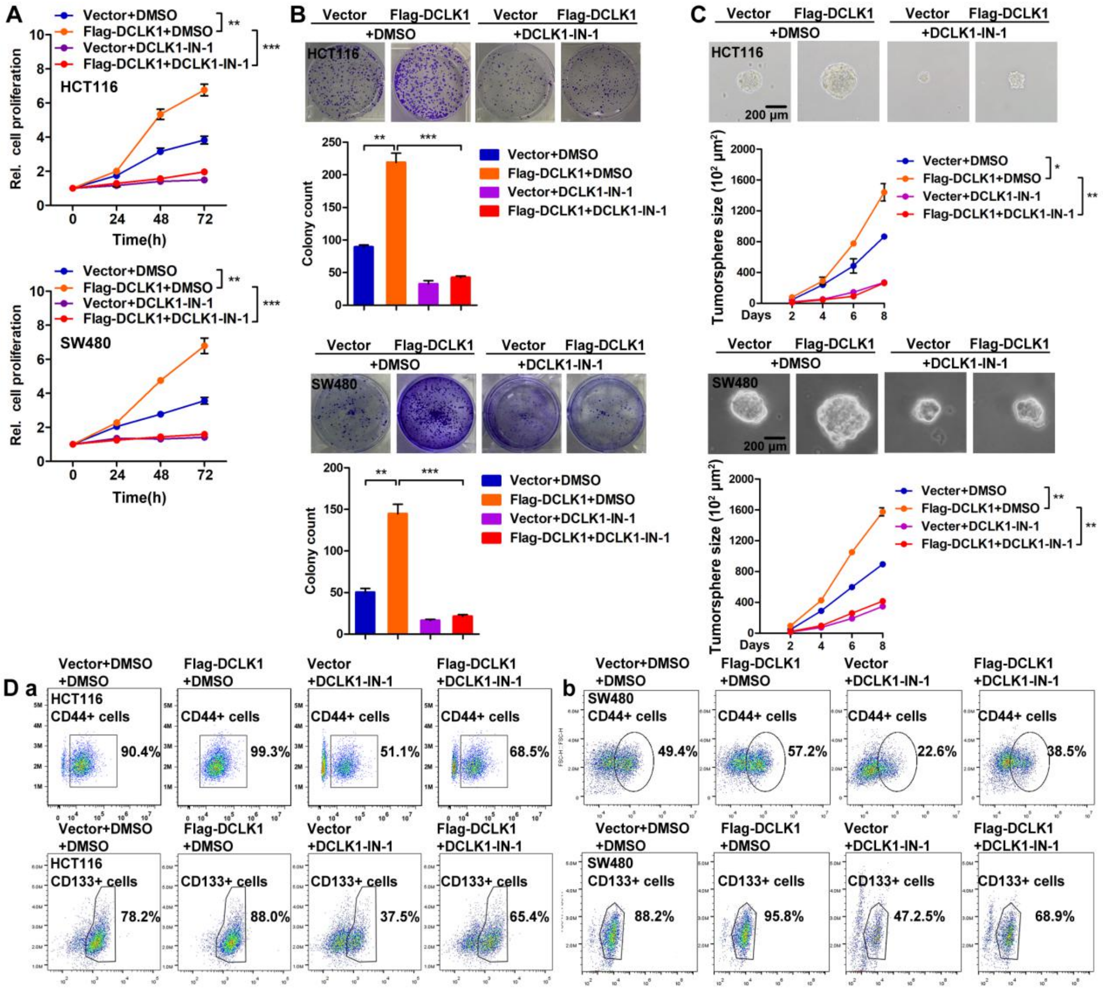

Figure S8. Targeting DCLK1 suppresses 5-fluorouracil resistant CRC cells. A. Cell proliferation of HCT116 and SW480 cells transfected with the indicated constructs or/and DCLK1-IN-1 was tested by CCK-8 assay (n=3). B. Colony formation ability of HCT116 and SW480 cells transfected with the indicated constructs for 48h and treated with DCLK1-IN-1 for 24h, was determined by clonogenic assay (n=3). C. Self-renewal activity of HCT116 and SW480 cells transfected with the indicated constructs for 48h and treated with DCLK1-IN-1 for 24h, was assessed by tumorsphere-forming assay (n=3). Scale bar represents 200 μm. D. CD44+ cells and CD133+ in HCT116 and SW480 cells transfected with the indicated constructs for 48h and treated with/without DCLK1-IN-1 for 24h, was

analysed by FACS assay. Data are expressed as mean ± SD. (*$P < 0.05$, ** $P < 0.01$, and *** $P < 0.001$.)

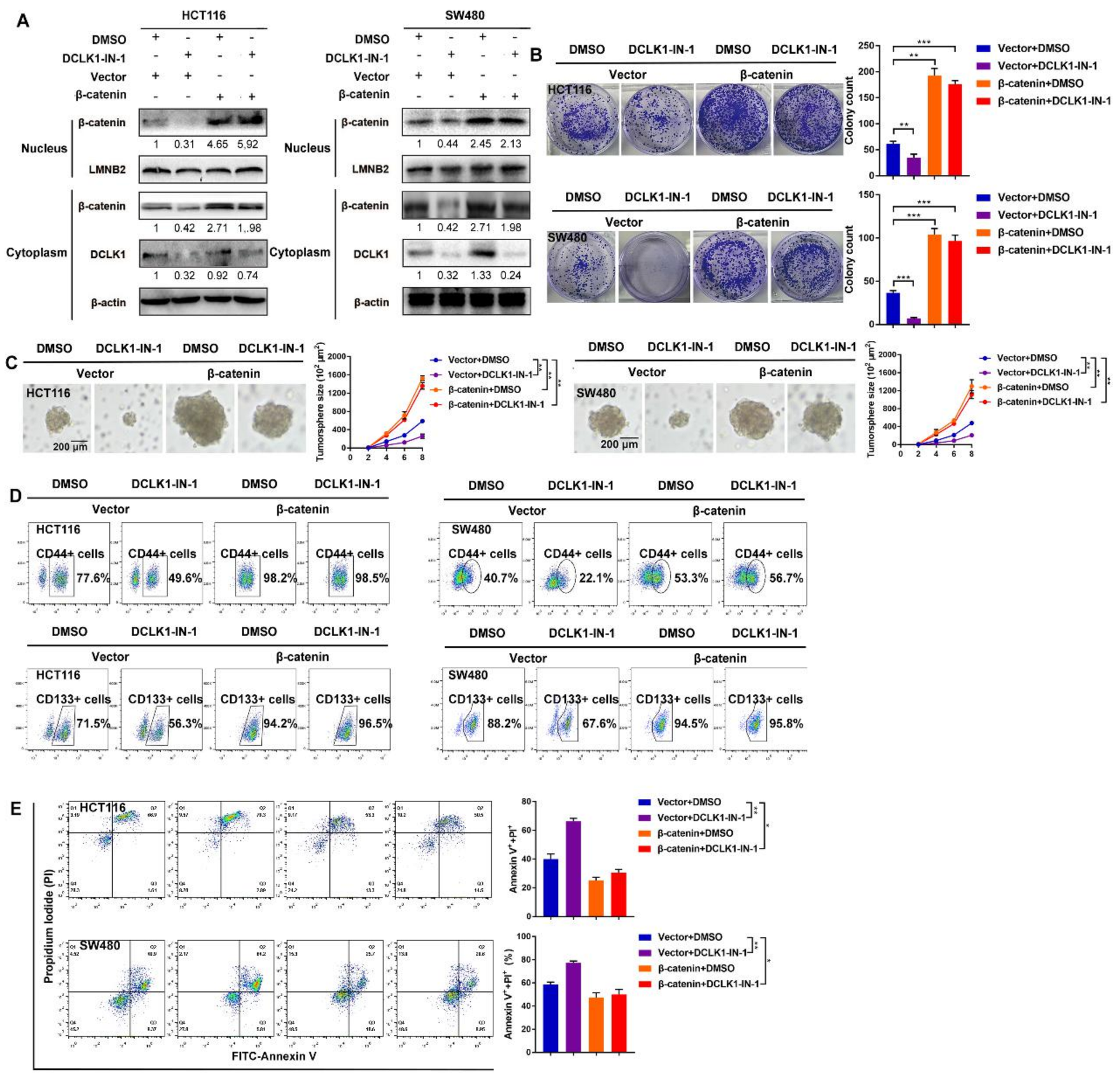

Figure S9. Targeting DCLK1 suppresses 5-fluorouracil resistant CRC cells. A. HCT116 and SW480 cells were transfected with the indicated constructs for 48h and treated with/without DCLK1 inhibitor (DCLK1-IN-1) for 24h. Protein in nucleus or cytoplasm was collected. Western blotting was conducted to detect the indicated protein level. B. Colony formation ability of HCT116 and SW480 cells transfected with the indicated constructs for 48h and treated with DCLK1-IN-1 for 24h, was determined by clonogenic assay (n=3). C. Self-renewal activity of HCT116 and SW480 cells transfected with the indicated constructs for 48h and treated with DCLK1-IN-1 for 24h, was assessed by tumorsphere-forming assay (n=3). Scale bar

represents 200 μm. D. CD44+ cells and CD133+ in HCT116 and SW480 cells transfected with the indicated constructs for 48h and treated with/without DCLK1-IN-1 for 24h, was analysed by FACS assay. E. Apoptosis of HCT116 and SW480 cells. Cells were transfected with the indicated constructs for 48h, and/or DCLK1-IN-1 for 24h, then subjected to FITC-Annexin V/propidium iodide staining and analyzed by FACS (n=3). Data are expressed as mean ± SD. (*P< 0.05, ** P< 0.01, and *** P< 0.001.)
