# Supplementary material for: Targeting DCLK1 overcomes 5‐fluorouracil resistance in colorectal cancer through inhibiting CCAR1/β‐catenin pathway‐mediated cancer stemness
Source: Clin Transl Med. 2022 May 6;12(5):e743. doi: 10.1002/ctm2.743 (PMC9076011; doi:10.1002/ctm2.743)
Supplement: Supplementary file 2 — Supporting Information [file CTM2-12-e743-s001.docx]

**Supplementary Table S1**

| **Supplementary Table S1. Primer sequences** | |
| --- | --- |
| Gene | Sequence |
| ACVRL1-F | TGGTTCCGGGAGACTGAGAT |
| ACVRL1-R | TGCTCGTGGTAGTGCGTGAT |
| ALPP-F | CCAGATGACTACAGCCAAGG |
| ALPP-R | GAGTCTCGGTGGATCTCGTAT |
| BMI-F | AAGTGACTCTGGGAGTGACAAGG |
| BMI-R | AGATTGGTGGTTACCGCTGG |
| CCAR1-F | CTTCGTTGGATGGACCAGAC |
| CCAR1-R | GATCAATGCCTGTCAGAGCCT |
| c-Myc-F | GCTGCCAAGAGGGTCAAGTT |
| c-Myc-R | GCTCCGTTTTAGCTCGTTCC |
| CST1-F | CCCAAGGAGGAGGATAGGAT |
| CST1-R | AAGTGAAGGGCACGCTGTAC |
| CYP11A1-F | AGACCTGGAAGGACCATGTG |
| CYP11A1-R | CTGTAGAGGATGCCACGGTAA |
| DCLK1-F | GTGATGACCAGGAGGTGCTT |
| DCLK1-R | CGCTGATCTACATCGACCAAC |
| EGR2-F | GCAAGTTCTCCATTGACCCTC |
| EGR2-R | GTGGTTGAAGCTGGGGAAGT |
| EPCAM-F | GCTCTGAGCGAGTGAGAACCTA |
| EPCAM-R | CTGAAGTGCAGTCCGCAAAC |
| EPHA4-F | GTGAGCCCTTGGAGGTTACA |
| EPHA4-R | ATTTACTCCGTCTCCGGCTG |
| IGFBP3-F | GTTGACTACGAGTCTCAGAGCAC |
| IGFBP3-R | CTACGGCAGGGACCATATTC |
| KLHDC8A-F | GACGATGGACGTGTTCGACA |
| KLHDC8A-R | CCAGGACAGTGGGTTGATTC |
| LGR5-F | CACCACATACCAGACTATGCC |
| LGR5-R | CCCAGGGAGTGGATTCTATTG |
| LOXL2-F | CACCCACTATGACCTGCTGA |
| LOXL2-R | GTCGATGTCATGGCGGTACA |
| Nanog-F | CTACCTACCCCAGCCTTTACT |
| Nanog-R | GGACTGGATGTTCTGGGTCT |
| PADI3-F | GGACATTTATGGGTCAGTGCC |
| PADI3-R | GCTGTCGTTGAGGTCATTGC |
| PLA2G4D-F | TGGGCTGACCTGTTGAGTGA |
| PLA2G4D-R | CGGTGAGCGTCTTGGTCTTA |
| PTPRZ1-F | CACTACAATCGCATAGGGACG |
| PTPRZ1-R | GGTGGCAGTTCAGTCACAGTCT |
| SAT1-F | TACCTATGACCCGTGGATTGG |
| SAT1-R | CTGATCCTATGCCAAAGCCTC |
| TBX4-F | CACTGCCTGAAAAGACGAGC |
| TBX4-R | GGCCCTGAACCTGAGTACAT |
